# Integrating patient-reported weight gain cause narratives into personalized obesity management: a data-driven approach with natural language processing and machine learning

**DOI:** 10.3389/fnut.2026.1777240

**Published:** 2026-04-14

**Authors:** Miksa M. Henkrich, Maitane Nuñez-Garcia, Ignacio Sajoux, Begoña de Cuevillas, Juan José López-Gómez, J. Alfredo Martínez, Daniel de Luis

**Affiliations:** 1 Precision Nutrition and Cardiometabolic Health Group, IMDEA Nutrition Institute, Campus of International Excellence (CEI) UAM+CSIC, Madrid, Spain; 2 Doctorate School of Food Sciences, Campus of International Excellence (CEI) UAM+CSIC, Autonomous University of Madrid, Madrid, Spain; 3 Department of Endocrinology and Nutrition, Medicine School, Center of Investigation of Endocrinology and Nutrition (IENVA), Hospital Clínico Universitario, University of Valladolid, Valladolid, Spain; 4 Medical Department, Pronokal Health Group, Barcelona, Spain; 5 Biomedical Research Centre for Obesity Physiopathology and Nutrition Network (CIBEROBN), Instituto de Salud Carlos III (ISCIII), Madrid, Spain; 6 Health Research Institute of Valladolid (IBioVALL), Valladolid, Spain

**Keywords:** artificial intelligence, medical nutritional—pharmacological therapy, obesity, obesity phenotypes, patient perspective, personalized nutrition, VLCKD—VLEKT, weight gain

## Abstract

**Background:**

Life course factors play an important role in the multifactorial etiology of obesity, yet quantitative analysis of patient-originated, unstructured weight gain cause narratives remains a challenge. This study automated the thematic labeling of such narratives with a large language model to assess the clinical relevance of patient-reported weight gain cause data in weight loss prediction and patient phenotyping.

**Subjects and methods:**

A total of 2,463 patients with overweight or obesity shared open weight gain cause narratives prior to starting a multidisciplinary medical-nutritional weight loss treatment, followed until reaching a pre-defined weight loss target or dropout. Narratives were labeled using 12 thematic categories in a GPT4.1 large language model. Associations of reported causal themes with age, sex, BMI class and treatment outcomes were evaluated using group-wise statistical comparisons and a Random Forest classifier. Weight gain cause co-occurrence patterns were modeled with a direct association network and pairwise risk ratio analyses. A partitional unsupervised clustering model integrating age, sex, baseline BMI and weight gain cause themes was designed to elucidate patient phenotypes defined by reported weight gain trajectories. Cluster-specific outcomes were compared using descriptive tests and linear mixed models.

**Results:**

Mean weight loss was 9.2 ± 6.8% over 108.6 ± 111.6 days. Automated weight gain narrative categorization achieved precision and recall of 0.906 and 0.897 against a reference sample. Reported weight gain causes were associated with age and sex but not BMI class. Associations between attributed causes and treatment outcomes were moderate, while between individual causes, strong associations were found. Disrupted schedules, mental health and external circumstances increased the risk ratio of unhealthy eating habits [3.65 (2.63–5.65), 2.16 (1.89–2.48), 1.51 (1.25–1.81) respectively], while medical issues and external circumstances increased physical inactivity risk [1.58 (1.31–1.90), 1.49 (1.23–1.82)]. Based on weight gain cause reports, age, sex and BMI class, seven clusters were identified with different demographic, clinical, treatment outcome and adherence characteristics.

**Conclusion:**

Patient-reported weight gain narrative analysis can be accurately automated using large language models, providing clinically relevant insights into obesity heterogeneity. While individual causes show modest associations with weight loss, their combined patterns allow the identification of distinct behavioral phenotypes with differential treatment responses. Integrating patient narratives into data-driven frameworks supports a more precise, person-centered obesity management.

## 1 Introduction

Obesity is a chronic, multifactorial, progressive disease process with serious health complications, placing a significant burden on individuals, healthcare systems and societies (1, 2). The multifactorial etiology of obesity leads to a phenotypic heterogeneity (3), a variability in individual responses to weight management interventions (4), and a need for personalized, precision approaches to its prevention and treatment (5).

Recent advancements in obesity research have made significant contributions to a better understanding of the role of genetic, metagenomic, metabolomic, behavioral and exposome factors, as well as socioeconomic and psychosocial circumstances in excess weight development (6–8), and several successful clinical trials have been reported with a personalized and precision approach to weight management (5). While there are increasingly more comprehensive theoretical models regarding obesity development, weight gain rarely happens as a solely biological process, isolated from a person’s individual and unique life history and experience (9). A considerable body of mainly qualitative, interview-based studies highlight the importance of subjective, circumstantial factors such as major life transitions, changes related to relationships, education or employment, loss of a family member or the use of food as a means of coping with past emotional trauma in the evolution of individual weight trajectories (9–12). As such, a recent position statement from The Obesity Society highlights a crucial need of addressing individual weight histories from a life course perspective in obesity management, and calls for prospective studies incorporating biopsychosocial and behavioral weight history data into obesity phenotyping (13).

In this context, the understanding and careful consideration of individual weight gain trajectories can highlight biological, psychosocial and behavioral mechanisms related to the development and maintenance of obesity, identify targets of intervention specific to the individual guiding downstream treatment (13), and can even have a therapeutic effect in and of itself, through the act of active, empathetic listening and the collaborative reshaping of weight-related internal narratives and identities (14, 15). This approach is a cornerstone of patient-centered care (16), nevertheless, current personalized and precision obesity management trends seem to lack an integration of weight histories and an insider perspective for the characterization, phenotyping and individualized management of obesities, especially when using quantitative, data-driven and prospective approaches. To the best of our knowledge, there is a clear scarcity of recent studies that quantitatively examine self-reported weight gain causes in large populations prior to the initiation of a weight loss intervention, particularly with the aim of evaluating how different weight gain trajectories influence subsequent treatment outcomes (17).

A potential bottleneck regarding the quantitative assessment of individual weight gain causes is, on one hand, the absence of standardized, universally accepted and practically adopted weight history assessment tools that could lay the foundation of data-driven workflows (13), and, on the other hand, the very personal, non-standard nature of weight histories, best captured through free-form or semi-structured narrative interactions. Personal narratives of weight gain cause attributions may reveal clinically and behaviorally actionable barriers to change or specific intervention targets, while being potentially impacted by intervention context, as well as recall, social desirability or internalized weight biases, often shaped by stigmatizing narratives of weight gain being a failure of personal responsibility (17, 18). As such, lay explanations of weight gain may not fully capture all etiological drivers, including natural biological variations of human body weight. Rather, they are best understood as windows to both patients’ individual life course circumstances as well as their relationship to their own health and bodies, which in itself carries clinical relevance, given that internalized weight bias was shown to negatively impact long-term weight maintenance (17). While interview-based assessments of weight-related narratives have shown to be impactful in individual clinical encounters (15), they are challenging to annotate, standardize and process at scale (19) in order to integrate weight history—and, potentially, attribution style—data into patient stratification, obesity phenotype discovery, treatment response prediction and treatment personalization.

Recent advancements in context-aware, highly flexible natural language processing workflows using large language models (LLMs), however, are allowing for new opportunities in the integration of free-text, narrative-based, patient-originated data in precision obesity research and management (20–22). Interest in the application of natural language processing algorithms in clinical and nutritional sciences has been growing since before the advent of large language models. Natural language processing based on conventional machine learning algorithms has been successfully applied to, for example, identify epidemiologically relevant dietary pattern shifts in a large population through the analysis of dietary intake surveys (23), or to analyze unstructured patient-reported outcomes in electronic health records (24). Nevertheless, transformer-based large language models have shown advantages over conventional, rule-based and non-neural machine learning algorithms even in early stages of their development (24). Compared to conventional, rule-based machine learning natural language processing workflows (25), LLMs based on the transformer architecture (26) have the advantage of retaining context and meaning in the text being processed, allowing for a more truthful, representative extraction of weight gain cause patterns from patient narratives (21). Such an application of LLMs can bridge the computational gap between rich, insightful and highly relevant yet unstructured, un-analyzeable patient-derived narrative data and standardized, high-dimensional clinical variables such as biochemical, omics or questionnaire-based lifestyle information.

Complementing the opportunities posed by LLMS, machine learning algorithms developed for classification, prediction and clustering tasks have gained particular traction in recent years regarding precision weight loss prediction and the discovery of obesity and metabolic health phenotypes. Nevertheless, when using machine learning models in a clinical setting where algorithm outputs influence treatment decisions, model interpretability is as important as predictive power (27). Explainable AI approaches are frameworks used in conjunction with machine learning models traditionally considered “black boxes,” in order to reveal the way in which a model arrived at a specific prediction. Feature importance measures, particularly novel, unified frameworks such as SHapley Additive exPlanations (SHAP) values, for example, can quantify the magnitude and direction of the influence of each predictor feature on a given prediction’s outcome, thus uncovering the mathematical process in which a model had arrived at that outcome (27). For example, a study by Choi et al. used SHAP values to identify how sentiments toward certain foods influence diet-related metabolic disease predictions (28). In summary, data-driven frameworks designed for interpretability are increasingly relevant in personalized nutrition for the stratified diagnosis and treatment of population subgroups with common physiological and behavioral traits in an integrated manner (29–31).

With regards to obesity management, current guidelines adopt multimodal frameworks ranging from behavioral, pharmacological and surgical interventions (32, 33). Structured multidisciplinary lifestyle interventions consisting of dietary, physical activity and behavior change recommendations generally produce 5–10% weight loss, however, weight regain remains a significant challenge (33, 34). Very low-calorie ketogenic diets (VLCKD) represent one of the most extensively studied nutritional approaches within lifestyle interventions and have been shown to induce rapid and substantial weight loss with good safety and tolerability when medically supervised (29, 35–37).

In recent years, based on accumulating VLCKD evidence, a new nomenclature has been proposed as very low-energy ketogenic therapy (VLEKT), highlighting key defining features such as dietary carbohydrate restriction, an adequate protein intake, a low intake in dietary fat, and the systematic use of micronutrient supplementation, together with a holistic, medically supervised approach, while preserving the original nutritional principles of the diet (37, 38).

In parallel with advances in lifestyle-based and precision nutrition approaches, pharmacological treatments of obesity have emerged as a key component of contemporary, multimodal weight management strategies (32, 33). Modern anti-obesity medications, including most recent GLP-1 and GIP agonists as of early 2026, target central and peripheral mechanisms regulating appetite, satiety, energy balance and glycemic control, and have demonstrated clinically meaningful and sustained weight loss across diverse patient populations (39, 40). Importantly, pharmacotherapy does not replace nutritional, physical activity or behavioral interventions, but rather offers an opportunity to enhance their effectiveness by reducing biological and neurobehavioral barriers to change (32). When embedded within structured lifestyle programs, pharmacological treatment may facilitate adherence, support early weight loss, and improve the durability of behavioral modifications, particularly in individuals with severe obesity, obesity-related comorbidities, or limited responses to lifestyle interventions alone (32).

From an integrative point of view, personalized and precision medicine frameworks need to be implemented in obesity management, considering individually varied food responses and other biological characteristics, as well as psycho-socio-economic contexts driving the manifestation of diverse obesity phenotypes and weight loss treatment responses (5, 8, 41–43). Applying a precision medicine perspective, the integration and individual tailoring of nutritional strategies, lifestyle modifications, behavioral consulting and, potentially, pharmacotherapy represents a promising avenue for individualized, synergistic treatment pathways that align biological susceptibility with patient-specific behavioral and psychosocial contexts (5, 41). As such, multi-modal obesity management based on lifestyle change with or without pharmacotherapy may benefit further from the clinical integration of patient-originated weight gain cause narratives.

Accordingly, this study aimed to systematically characterize the prevalence, distribution, co-occurrence patterns and clinical relevance of patient-reported weight gain causes in a large cohort undergoing a multidisciplinary, VLEKT-based weight loss intervention in a real-world setting, using large language models to transform unstructured narrative data into structured variables. Furthermore, by integrating these data points into a machine learning–based, unsupervised phenotyping framework, this study identifies distinct behavioral patient profiles with differential weight loss responses, and discusses clinically actionable insights to support patient-centered, personalized and precision obesity management.

## 2 Subjects and methods

### 2.1 Study settings

This study analyzed data from two large prospective, observational, multi-centric, real clinical practice patient cohorts (PROMET CONNECT and PROMET CONNECT GEN studies) voluntarily undergoing obesity treatment with a commercially available multidisciplinary, stepped lifestyle intervention for weight loss (PronoKal Method^®^, Pronokal Health Group, Barcelona, Spain) in a routine clinical setting. The intervention consists of dietary, physical activity, nutritional coaching and digital weight monitoring components, described elsewhere in detail (29). Briefly, patients follow a VLEKT for rapid initial weight loss, followed by a balanced hypocaloric diet. Stepped physical activity recommendations, nutritional-behavioral coaching sessions for emotional support, and an app-based weight and body composition monitoring also form part of the program, whose components are tailored to patients’ individual characteristics, engagement and preferences.

Patient participation was conditioned to written informed consent, adult age, presence of overweight or obesity as per baseline BMI ≥ 25 kg/m^2^, and the absence of pregnancy, lactation and cardiovascular, metabolic, oncological, renal, hepatic, psychiatric or endocrine comorbidities except for type II diabetes mellitus. Recruitment was ongoing between January 2020 and July 2024 at several Spanish, Portuguese, Italian, Belgian and Swiss clinical centers by medical doctors prescribing the commercially available intervention. Patients consented to the research use of all of their collected clinical data, retaining full rights to withdraw consent, as well as to access, withdraw or modify stored information. The two cohort studies were coordinated by the Department of Endocrinology and Nutrition of the University Hospital of Valladolid, Spain. The studies were conducted in accordance with the Declaration of Helsinki and received ethical approval from the Medical Investigation Ethics Committee of East Valladolid, Valladolid, Spain (PROMET CONNECT: PI 20–1936; PROMET CONNECT GEN: PI 21–2,299).

### 2.2 Data collection

Longitudinal body weight and body composition data was continuously recorded by patients through digital bioimpedance scales (44) (LifeVit BL2500, LifeVit, Barcelona, Spain) connected to a mobile application (PronoKal Connect^®^, Pronokal Health Group), available at-home as part of the intervention. Sociodemographic, baseline clinical and longitudinal treatment administration-related data were recorded by the medical team through a centralized digital platform. As part of their first visit, patients were asked about the perceived causes of their weight gain prior to the start of the intervention. A total of 2,463 patients compliant with inclusion criteria shared unstructured verbal narratives in their mother tongue that were recorded digitally by registered dietitians through the same platform. Data collected anonymously through the aforementioned digital channels was stored in a centralized electronic database maintained by Pronokal Health Group. Following standard outlier removal, namely, exclusion of clinically implausible values, de-identified data from consenting patients were encrypted and transferred to the academic researcher team, who conducted subsequent data handling and analysis independently with no sponsor oversight. In this investigation, the following variables were included: sex, age at baseline, weight gain cause narrative, longitudinal body weight and body composition measurements.

### 2.3 Natural language processing of weight gain cause data

Original, unstructured weight gain cause narratives were first read iteratively by two authors in their original language to identify and reach consensus on common themes (19). Narratives provided varying level of detail and complexity on self-attributed drivers of weight gain, with 12 recurring themes being identified as the most common by authors: weight gain due to women’s health issues or pregnancy; mental health issues; family issues; eating habits; physical inactivity; injuries, medication side effects or comorbidities; lockdowns during the COVID-19 pandemic; schedule problems; lifestyle and external circumstances; smoking cessation; weight regain after previous weight loss success; or none of the above in case of vague, unspecific causality narratives.

Posterior to human audit, to label individual narratives with one or more corresponding, self-evident thematic labels at scale, the context-aware natural language processing capabilities of a LLM were harnessed (20). After an iterative testing of several models, category label wordings and prompt structures, GPT4.1, a non-reasoning OpenAI model was elected due to its high instruction adherence and large context window (45). Temperature, controlling model output randomness, was set to 1, permitting a modest level of flexibility to better contextualize challenging cases (46). Further details on model selection and testing are available in Supplementary File S1. GPT4.1 was accessed through OpenAI’s application programming interface (API) with a custom Python script using a few-shot prompt structure (47) with stringent instructions and real-life examples (Supplementary File S1). To ensure data privacy, only weight gain cause narratives were accessed by the API, with assigned category labels re-linked to patient identifiers and clinical variables locally.

To evaluate the LLM’s categorization accuracy, a randomly selected sample of 300 narratives were labeled by 3 independent, blinded researchers following the instructions given to the LLM in the prompt (Supplementary File S1). Initial inter-rater agreement was established at Krippendorff’s *α* = 0.834, and disagreements were resolved through discussion to establish a consensus reference set against which the LLM output was evaluated quantifying both category-level and average precision, recall and F1 scores, as well as case-level exact matches (Supplementary Data S1; Supplementary File S1). A complementary qualitative audit further revised the nature of mistakes made by the mode (Supplementary Data S1).

Independently of categorization, with the goal of facilitating transparent data reporting, narratives were translated to English using GPT4o-mini, a light-weight OpenAI model (48) (Supplementary File S1; Supplementary Data S1).

### 2.4 Statistical and machine learning analyses

Data processing and analysis was conducted in Python v3.12.3 in a Kiro v1.21.0 integrated development environment. First, patients were stratified by demographic groups as sex, age (under or over the median age of 46 years), BMI (under or over 30 kg/m^2^) and by the presence of weight gain causes. Baseline characteristics and treatment outcomes were compared between demographic strata, while weight gain cause groups were compared to the population mean to assess the statistical significance of group-wise differences. Given the frequency of non-normal distributions, a Mann–Whitney U test was used for continuous and a Chi squared or Fisher’s exact test (49) was applied for categorical variable comparisons. *p*-values were adjusted for multiple testing using the Benjamini-Hochberg procedure (50).

The influence of reported weight gain causes on weight loss was assessed as the change in the likelihood of 10% weight loss achievement by weight gain cause. First, risk differences (51) were calculated for the outcome by attributed weight gain cause groups and displayed on a forest plot using *matplotlib* (52). Subsequently, a Random Forest classifier predicting the likelihood of 10% weight loss by weight gain cause themes, adjusted for age, sex and baseline BMI, was trained on the data with 5-fold cross-validation on a 70/30 train-test split, and feature importances were extracted as proxies for influence in weight loss. Permutation feature importance scores were calculated on the test set by quantifying the impact of randomly shuffling predictor values on the model’s predictive performance (53). SHapley Additive exPlanations (SHAP) values, a game-theory based explainable AI metric were extracted and visualized using the *shap* library to quantify the contribution of each feature—here, weight gain causes—to the prediction (27).

Associations between individual weight gain cause themes were examined using an Ising model. Originating in statistical physics, the model is increasingly applied in health and psychology research to infer conditional co-occurrence patterns among binary variables of a network, while accounting for the presence of all other variables using regularized regression (54). In this study, direct conditional associations between weight gain causes, i.e., the co-occurrence of specific cause narratives beyond what is explained by the presence of other causes, was estimated with an Ising network using the *IsingFit* R package, with *rpy2* and *networkx* for Python integration and visualization (55–57). To complement the network model by approximating the strength and directionality of associations between weight gain cause themes, i.e., to estimate how the presence of one cause increases the likelihood of another, pairwise risk ratios (51) were calculated between weight gain causes and plotted with *seaborn*.

Subsequently, a behavioral patient phenotyping framework was designed integrating age, sex, baseline BMI and attributed weight gain cause data in an unsupervised clustering model. After thorough consideration and testing of several clustering approaches (Supplementary Data S2), a k-medoids algorithm was run on a hybrid distance matrix calculating demographic and narrative-based patient dissimilarities in two separate steps (58). A Gower dissimilarity matrix, adept at handling mixed continuous and binary data (59), was built on age, sex and baseline BMI variables of the cohort. Patient similarities based on binary weight gain cause themes were calculated using the Jaccard index, quantifying the number of common causes between two individuals (58). The distance matrices were hybridized with a weight of 0.5 each to give equal importance to the “demographic” and “weight gain cause” variable blocks during clustering. K-medoids clustering, an analogue to the K-means method that uses actual patients’ data over statistical means during cluster initialization (60), was applied with a partitioning around medoids algorithm and a k-medoids++ initialization iterated over 10 random seeds and settling on the most optimal one to ensure rigorous and non-random partitioning of data (61). A comprehensive evaluation of clustering solutions (Supplementary Data S2) relied on the Silhouette, Davies-Bouldin and Calinski-Harabasz indices, the clinical relevance and interpretability of clusters, and the significance of overall between-cluster differences as detected by a Kruskal-Wallis test (62).

A final number of *k* = 7 clusters were statistically compared to the population mean with a Mann–Whitney U or a Chi^2^/Fisher’s exact test as in the case of weight gain cause group-wise comparisons, with multiple testing corrections applied. *Matplotlib* and *seaborn* were used to display cluster-wise weight gain cause theme prevalences plotted on a heatmap, and clinical variable distributions using stacked bar charts for binary and split violin plots for continuous variables. A comprehensive overview of cluster-wise deviations from the population mean across all clinical variables were plotted on a lollipop plot as percent change differences, i.e., % change = ((cluster mean or % - population mean or %)/population mean or %) * 100 with *p*-values extracted from the cluster-versus-population comparisons. Moreover, cluster-specific mean weight change trajectories were estimated with linear mixed models in *statsmodels* over a 1-year period, acknowledging that the population-level mean follow-up length of 108.6 ± 111.6 days resulted in sparse long-term follow-up data for modeling.

A visual summary of the study’s data collection and analysis workflow is available in Supplementary Figure S1.

## 3 Results

The study population, with 81.9% of women, a mean age of 46.2 ± 10.5 years and a mean baseline BMI of 30.1 ± 3.0, achieved 9.2 ± 6.8% weight loss over 108.6 ± 111.6 days on average (Table 1). Men, as well as patients with a younger age and baseline overweight showed faster weight loss and shorter follow-ups, while the magnitude of total weight loss only differed by baseline BMI (Table 1).

**Table 1:** Comparison of age, sex and baseline BMI groups in the study population, data is presented as N (%) for continuous and mean ± SD for categorical variables.

Variable	Overall population, *N* = 2,463	Age < median (46 years), *N* = 1,140	Age ≥ median (46 years), *N* = 1,323	Age: *p*-value	Men, *N* = 445	Women, *N* = 2,018	Sex: *p*-value	BMI < 30 kg/m^2^, *N* = 1,253	BMI ≥ 30 kg/m^2^, *N* = 1,210	BMI: *p*-value
Demographics and baseline anthropometry										
Sex (% of women)	2018 (81.9%)	941 (82.5%)	1,077 (81.4%)	0.610	0 (0.0%)	2018 (100.0%)	**0.000**	1,100 (87.8%)	918 (75.9%)	**0.000**
Age (years)	46.18 ± 10.54	37.22 ± 6.38	53.90 ± 6.55	**0.000**	46.46 ± 10.81	46.12 ± 10.48	0.511	46.08 ± 10.55	46.28 ± 10.53	0.489
Baseline weight (kg)	82.56 ± 12.25	82.96 ± 12.36	82.22 ± 12.16	0.188	98.53 ± 13.29	79.04 ± 8.70	**0.000**	75.12 ± 7.84	90.26 ± 11.20	**0.000**
Baseline BMI (kg/m^2^)	30.06 ± 2.96	29.98 ± 2.99	30.12 ± 2.93	0.336	31.79 ± 3.46	29.68 ± 2.69	**0.000**	27.62 ± 1.40	32.58 ± 1.82	**0.000**
Treatment outcomes										
Follow-up length (days)	108.57 ± 111.61	104.85 ± 114.64	111.78 ± 108.86	**0.023**	92.40 ± 100.69	112.14 ± 113.59	**0.000**	100.16 ± 106.68	117.29 ± 115.89	**0.000**
Total weight loss (%)	−9.21 ± 6.85	−9.30 ± 6.95	−9.14 ± 6.77	0.844	−9.42 ± 6.61	−9.17 ± 6.91	0.224	−8.30 ± 5.99	−10.16 ± 7.53	**0.000**
BMI reduction (kg/m^2^)	−2.79 ± 2.18	−2.81 ± 2.20	−2.78 ± 2.17	0.862	−3.06 ± 2.30	−2.73 ± 2.15	**0.005**	−2.29 ± 1.69	−3.31 ± 2.50	**0.000**
60-day dropouts (n)	1,080 (43.8%)	538 (47.2%)	542 (41.0%)	**0.006**	213 (47.9%)	867 (43.0%)	0.067	590 (47.1%)	490 (40.5%)	**0.002**
Achieved 10% weight loss (n)	1,186 (48.2%)	540 (47.4%)	646 (48.8%)	0.610	221 (49.7%)	965 (47.8%)	0.514	565 (45.1%)	621 (51.3%)	**0.004**
Days to 10% weight loss	52.59 ± 31.92	49.50 ± 29.68	55.17 ± 33.48	**0.000**	39.60 ± 17.66	55.57 ± 33.67	**0.000**	50.85 ± 31.45	54.18 ± 32.29	0.052
Self-reported causes of weight gain										
Women’s health and pregnancy	652 (26.5%)	340 (29.8%)	312 (23.6%)	**0.002**	10 (2.2%)	642 (31.8%)	**0.000**	355 (28.3%)	297 (24.5%)	0.056
Mental health	594 (24.1%)	284 (24.9%)	310 (23.4%)	0.545	74 (16.6%)	520 (25.8%)	**0.000**	309 (24.7%)	285 (23.6%)	0.608
Family issues	129 (5.2%)	44 (3.9%)	85 (6.4%)	**0.012**	7 (1.6%)	122 (6.0%)	**0.000**	59 (4.7%)	70 (5.8%)	0.361
Medication, disease or injury	402 (16.3%)	147 (12.9%)	255 (19.3%)	**0.000**	61 (13.7%)	341 (16.9%)	0.115	201 (16.0%)	201 (16.6%)	0.774
Physical inactivity	558 (22.7%)	262 (23.0%)	296 (22.4%)	0.844	162 (36.4%)	396 (19.6%)	**0.000**	277 (22.1%)	281 (23.2%)	0.607
Eating habits	822 (33.4%)	394 (34.6%)	428 (32.4%)	0.385	187 (42.0%)	635 (31.5%)	**0.000**	430 (34.3%)	392 (32.4%)	0.428
Schedule	147 (6.0%)	69 (6.1%)	78 (5.9%)	0.937	38 (8.5%)	109 (5.4%)	**0.016**	70 (5.6%)	77 (6.4%)	0.547
Smoking cessation	143 (5.8%)	41 (3.6%)	102 (7.7%)	**0.000**	17 (3.8%)	126 (6.2%)	0.062	75 (6.0%)	68 (5.6%)	0.777
Treatment discontinuation or relapse	318 (12.9%)	146 (12.8%)	172 (13.0%)	0.937	48 (10.8%)	270 (13.4%)	0.162	160 (12.8%)	158 (13.1%)	0.878
COVID-19 pandemic	314 (12.7%)	148 (13.0%)	166 (12.5%)	0.857	56 (12.6%)	258 (12.8%)	0.971	150 (12.0%)	164 (13.6%)	0.361
External circumstances	381 (15.5%)	214 (18.8%)	167 (12.6%)	**0.000**	74 (16.6%)	307 (15.2%)	0.499	200 (16.0%)	181 (15.0%)	0.606
None of the above	276 (11.2%)	121 (10.6%)	155 (11.7%)	0.545	77 (17.3%)	199 (9.9%)	**0.000**	133 (10.6%)	143 (11.8%)	0.463

*p*-values were calculated using Mann–Whitney U test for continuous variables; Chi^2^ test for categorical variables; and Fisher’s exact test for categorical variables with a low expected variance count. *p*-values were adjusted for multiple comparisons using the Benjamini-Hochberg method.*P* values < 0.05 are highlighted in bold.

The automated thematic labeling of weight gain cause narratives into 12 predefined categories using the GPT-4.1 engine achieved macro-averaged precision, recall and F1 score values of 0.906, 0.897, and 0.897, respectively, with case-level exact matches of 81.0% (Supplementary Data S1; Supplementary File S1), when evaluated against a subset of 300 consensus coded reference narratives. Complete mismatches were only found in 2.3% of cases, exclusively related to the “None of the above” category label. In descriptive analyses stratified by demographic factors, several weight gain cause themes showed statistically significant associations with age and sex, whereas no consistent associations were observed with baseline BMI (Table 1). Weight gain attributed to family-related issues, medical factors, or smoking cessation was more frequently reported by older patients, while causes related to women’s health and external circumstances were more prevalent among younger individuals. Women more commonly described biological life transitions and psychosocial challenges as drivers of weight gain, whereas men more often referred to health-related behaviors and scheduling constraints or did not mention specific causes. Although baseline BMI was not significantly associated with reported weight gain causes, higher baseline body weight was observed among patients mentioning physical inactivity or vague, non-specific weight gain narratives (Table 2). In contrast, weight gain attributed to women’s health issues was inversely associated with baseline body weight.

**Table 2:** Comparison of weight gain cause groups to the population average.

Variable	Overall population, *N* = 2,463	Women’s health and pregnancy, *N* = 652	Mental health, *N* = 594	Family issues, *N* = 129	Medication, disease or injury, *N* = 402	Physical inactivity, *N* = 558	Eating habits, *N* = 822	Schedule, *N* = 147	Smoking cessation, *N* = 143	Treatment discontinuation or relapse, *N* = 318	COVID-19 pandemic, *N* = 314	External circumstances, *N* = 381	None of the above, *N* = 276
Demographics and baseline anthropometry													
Sex (% of females)	2018 (81.9%)	642 (98.5%)**	520 (87.5%)**	122 (94.6%)**	341 (84.8%)	396 (71.0%)**	635 (77.3%)**	109 (74.1%)*	126 (88.1%)	270 (84.9%)	258 (82.2%)	307 (80.6%)	199 (72.1%)**
Age (years)	46.18 ± 10.54	45.14 ± 9.58**	45.94 ± 9.90	49.37 ± 9.82**	47.87 ± 10.25**	45.67 ± 10.78	45.72 ± 10.68	45.53 ± 10.13	50.29 ± 8.70**	46.67 ± 10.57	46.59 ± 10.60	43.51 ± 11.09**	46.99 ± 10.51
Baseline weight (kg)	82.56 ± 12.25	79.13 ± 9.03**	81.40 ± 10.67	81.95 ± 10.15	82.32 ± 11.89	84.64 ± 13.27**	83.12 ± 12.17	83.85 ± 12.46	80.91 ± 10.04	82.63 ± 12.67	82.32 ± 11.84	82.80 ± 12.03	85.16 ± 14.44**
Baseline BMI (kg/m^2^)	30.06 ± 2.96	29.70 ± 2.73*	29.97 ± 2.92	30.28 ± 2.54	30.20 ± 2.85	30.22 ± 2.98	30.06 ± 2.95	30.17 ± 3.01	29.90 ± 2.84	30.22 ± 3.07	30.20 ± 2.98	29.94 ± 2.84	30.25 ± 3.20
Treatment outcomes													
Follow-up length (days)	108.57 ± 111.61	109.09 ± 115.94	111.13 ± 105.98	118.19 ± 119.12	108.13 ± 115.24	118.54 ± 114.55*	109.56 ± 115.85	118.85 ± 123.39	112.58 ± 124.28	120.21 ± 119.06	126.87 ± 120.50*	110.52 ± 108.01	101.37 ± 113.48
Total weight loss (%)	−9.21 ± 6.85	−9.33 ± 6.79	−9.28 ± 6.84	−9.56 ± 7.44	−8.86 ± 6.85	−9.62 ± 6.93	−9.07 ± 6.94	−9.02 ± 6.55	−9.61 ± 6.71	−9.28 ± 6.45	−9.97 ± 6.87	−8.79 ± 6.70	−9.02 ± 6.85
BMI reduction (kg/m^2^)	−2.79 ± 2.18	−2.77 ± 2.09	−2.80 ± 2.14	−2.89 ± 2.32	−2.70 ± 2.21	−2.94 ± 2.20	−2.75 ± 2.21	−2.70 ± 2.03	−2.91 ± 2.16	−2.81 ± 2.06	−3.03 ± 2.22*	−2.64 ± 2.09	−2.79 ± 2.25
60-day dropouts (n)	1,080 (43.8%)	289 (44.3%)	246 (41.4%)	50 (38.8%)	174 (43.3%)	218 (39.1%)*	362 (44.0%)	58 (39.5%)	71 (49.7%)	120 (37.7%)*	125 (39.8%)	171 (44.9%)	139 (50.4%)*
Achieved 10% weight loss (n)	1,186 (48.2%)	314 (48.2%)	292 (49.2%)	64 (49.6%)	172 (42.8%)	296 (53.0%)*	394 (47.9%)	80 (54.4%)	69 (48.3%)	159 (50.0%)	169 (53.8%)	182 (47.8%)	125 (45.3%)
Days to 10% weight loss	52.59 ± 31.92	54.49 ± 28.00*	53.27 ± 26.96	56.20 ± 26.17	55.46 ± 35.58	50.47 ± 37.29*	52.12 ± 36.91	49.06 ± 18.56	50.64 ± 27.94	55.89 ± 26.33*	53.77 ± 25.11	54.12 ± 36.77	52.81 ± 31.24
Self-reported causes of weight gain													
Women’s health and pregnancy	652 (26.5%)	652 (100.0%)**	136 (22.9%)	26 (20.2%)	103 (25.6%)	94 (16.8%)**	148 (18.0%)**	29 (19.7%)	43 (30.1%)	77 (24.2%)	64 (20.4%)*	62 (16.3%)**	0 (0.0%)**
Mental health	594 (24.1%)	136 (20.9%)	594 (100.0%)**	66 (51.2%)**	91 (22.6%)	128 (22.9%)	309 (37.6%)**	54 (36.7%)**	18 (12.6%)**	102 (32.1%)**	75 (23.9%)	103 (27.0%)	0 (0.0%)**
Family issues	129 (5.2%)	26 (4.0%)	66 (11.1%)**	129 (100.0%)**	19 (4.7%)	14 (2.5%)**	45 (5.5%)	11 (7.5%)	10 (7.0%)	24 (7.5%)	17 (5.4%)	27 (7.1%)	0 (0.0%)**
Medication, disease or injury	402 (16.3%)	103 (15.8%)	91 (15.3%)	19 (14.7%)	402 (100.0%)**	127 (22.8%)**	103 (12.5%)*	17 (11.6%)	9 (6.3%)**	60 (18.9%)	52 (16.6%)	37 (9.7%)**	0 (0.0%)**
Physical inactivity	558 (22.7%)	94 (14.4%)**	128 (21.5%)	14 (10.9%)**	127 (31.6%)**	558 (100.0%)**	237 (28.8%)**	44 (29.9%)	11 (7.7%)**	67 (21.1%)	97 (30.9%)**	116 (30.4%)**	2 (0.7%)**
Eating habits	822 (33.4%)	148 (22.7%)**	309 (52.0%)**	45 (34.9%)	103 (25.6%)**	237 (42.5%)**	822 (100.0%)**	95 (64.6%)**	22 (15.4%)**	124 (39.0%)	105 (33.4%)	164 (43.0%)**	0 (0.0%)**
Schedule	147 (6.0%)	29 (4.4%)	54 (9.1%)*	11 (8.5%)	17 (4.2%)	44 (7.9%)	95 (11.6%)**	147 (100.0%)**	2 (1.4%)*	25 (7.9%)	19 (6.1%)	36 (9.4%)*	0 (0.0%)**
Smoking cessation	143 (5.8%)	43 (6.6%)	18 (3.0%)*	10 (7.8%)	9 (2.2%)**	11 (2.0%)**	22 (2.7%)**	2 (1.4%)*	143 (100.0%)**	10 (3.1%)	23 (7.3%)	11 (2.9%)*	0 (0.0%)**
Treatment discontinuation or relapse	318 (12.9%)	77 (11.8%)	102 (17.2%)*	24 (18.6%)	60 (14.9%)	67 (12.0%)	124 (15.1%)	25 (17.0%)	10 (7.0%)	318 (100.0%)**	45 (14.3%)	65 (17.1%)*	2 (0.7%)**
COVID-19 pandemic	314 (12.7%)	64 (9.8%)*	75 (12.6%)	17 (13.2%)	52 (12.9%)	97 (17.4%)**	105 (12.8%)	19 (12.9%)	23 (16.1%)	45 (14.2%)	314 (100.0%)**	58 (15.2%)	1 (0.4%)**
External circumstances	381 (15.5%)	62 (9.5%)**	103 (17.3%)	27 (20.9%)	37 (9.2%)**	116 (20.8%)**	164 (20.0%)**	36 (24.5%)*	11 (7.7%)*	65 (20.4%)*	58 (18.5%)	381 (100.0%)**	0 (0.0%)**
None of the above	276 (11.2%)	0 (0.0%)**	0 (0.0%)**	0 (0.0%)**	0 (0.0%)**	2 (0.4%)**	0 (0.0%)**	0 (0.0%)**	0 (0.0%)**	2 (0.6%)**	1 (0.3%)**	0 (0.0%)**	276 (100.0%)**

Data is presented as N (%) for continuous and mean ± SD for categorical variables. Weight gain cause groups were compared to the population average (Column 2) using Mann–Whitney U test for continuous variables; Chi^2^ test for categorical variables; and Fisher’s exact test for categorical variables with a low expected variance count. *raw *p* > 0.05. **p > 0.05 after adjusting for multiple comparisons using the Benjamini-Hochberg method.

Regarding the associations of individual weight gain causes with intervention adherence and outcomes, follow-up length often deviated from the population mean in different weight gain cause groups with up to 18 days of difference on average. Dropout rates at 2 months differed up to 7 percentage points from the population average in some weight gain cause groups (Table 2). However, observed deviations were at best tending toward statistical significance when controlling for false discovery rates (Table 2). Similarly, while weight gain cause group-wise differences were observed in total weight loss, BMI reduction, likelihood and speed of achieving 10% weight loss, these differences rarely reached statistical significance (Table 2).

**Figure 1:**
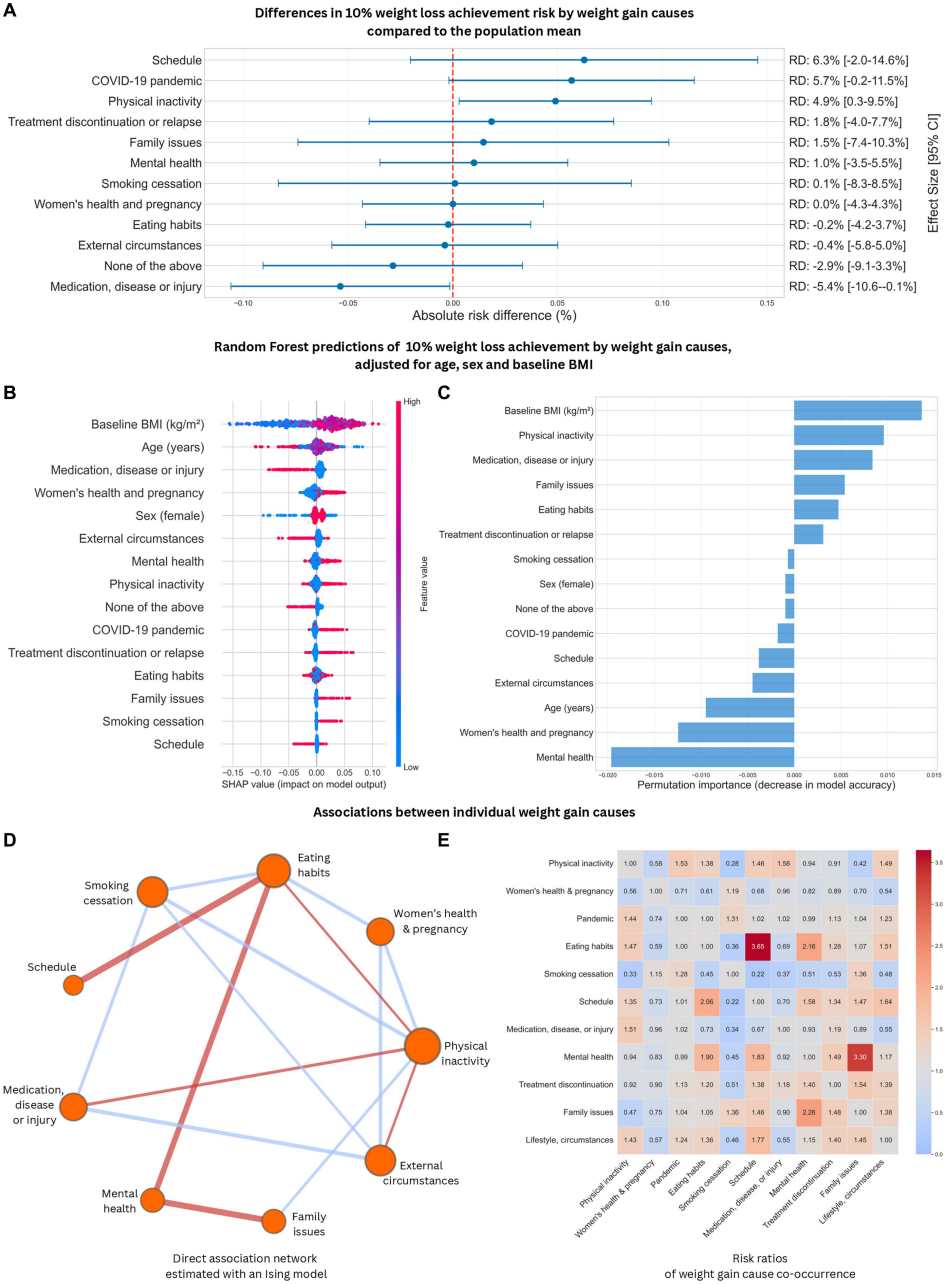
Analysis weight gain causes: impact on weight loss by weight gain cause and interrelationships between individual causes. Weight loss impact was assessed as: risk differences in 10% weight loss achievement between weight gain cause groups and the whole population **(A)**; and variable contributions to Random Forest predictions of the likelihood of 10% weight loss evaluated through permutation feature importance **(B)** and SHAP **(C)** values. On panel B, SHAP values indicate both the magnitude and directionality of a feature’s influence on a prediction, in function of feature value. Features are ordered along the *y* axis by mean absolute SHAP value. Dots represent individual predictions, colors represent feature values, which are 0/1 for binary-encoded weight gain causes and sex, where 1 = female sex or cause present. For example, presence of the “medication, disease or injury” label generally decreased the likelihood of 10% weight loss in predictions, while presence of “physical inactivity” often, but not always, increased it. On Panel C, permutation feature importance values quantify the likelihood of a feature being needed to make a specific prediction, independent of feature value. Features with positive permutation feature importance values caused meaningful improvements to prediction accuracy. Associations between weight gain causes were assessed with a direct association network based on the Ising model **(D)** where nodes are weight gain causes, red edges represent trends of co-occurrence, blue edges represent trends of mutual exclusivity, edge thickness represents association strength. A complementary risk ratio heatmap **(E)** indicates the probability of the occurrence of weight gain cause Y (*y* axis) given the presence of weight gain cause X (*x* axis). Red cells indicate co-occurrence, blue cells signal mutual exclusivity.

To examine the influence of individual weight gain cause attributions on weight loss in greater detail, we devised risk difference and random forest analyses with the outcome of 10% weight loss achievement. Figure 1A shows statistically significant risk increases for this outcome when weight gain is attributed to physical inactivity (risk differences with 95% confidence intervals: 4.9% (0.3–9.5%), while the inverse is true for weight gain related to medical factors [−5.4% (−10.6 to −0.1%)]. A random forest classifier with permutation feature importance and SHAP value analyses adjusted for age, sex and baseline BMI, provided further insight by analyzing the contribution of each weight gain cause variable to a machine learning-based weight loss prediction. While the model’s overall predictive power based on these variables alone was moderate, feature importance and variable contribution analyses provide an insight into the potential direction and magnitude of the impact of reported weight gain cause variables on clinical outcomes (Figures 1B,C; Supplementary Figure S2). Permutation feature importance analysis reinforces the influence of physical inactivity and medical issues—such as injuries and iatrogenesis—on weight loss outcomes (Figure 1B), while the SHAP beeswarm plot (Figure 1C) suggests a consistent, often positive directionality in the influence of most weight gain causes on weight loss, meaning that the model interprets most weight gain causes as linked to higher likelihoods of 10% weight loss achievement.

In order to overview the associations of reported weight gain causes to each other and identify co-occurring or cumulative drivers of weight gain, a direct correlation network analysis complemented with a risk ratio heatmap was applied (Figures 1D,E). Accordingly, weight gain attributed to eating habits showed positive associations with weight gain reportedly driven by schedules, mental health issues, physical inactivity and external circumstances, with the associations to schedules and mental health challenges being the strongest (Figure 1D). Reporting eating habits if schedule issues were present was 3.65 (95% confidence interval: 2.63–5.65) times more likely, while the risk of mentioning eating habits given the presence of mental health problems was 2.16 (1.89–2.48) higher (Figure 1E). Mental health problems, in turn, were strongly associated with family issues [RR: 3.30 (2.36–4.60)]. Physical inactivity and external circumstances increased the risk of reported unhealthy eating habits with ratios of 1.47 (1.27–1.70) and 1.51 (1.25–1.81), respectively. Risk for a lack of physical inactivity was found to be 1.58 (1.31–1.90) times higher in the presence of medical issues such as injuries, and 1.49 (1.23–1.82) times higher with reference to external circumstances.

Finally, to identify and characterize patient behavioral phenotypes as defined by reported weight gain cause themes, age, sex and baseline BMI, a K-medoids clustering approach was designed. After a thorough evaluation of candidate clusters according to clustering quality indices and clinical criteria (Supplementary Data S2), *k* = 7 clusters were deemed optimal.

**Figure 2:**
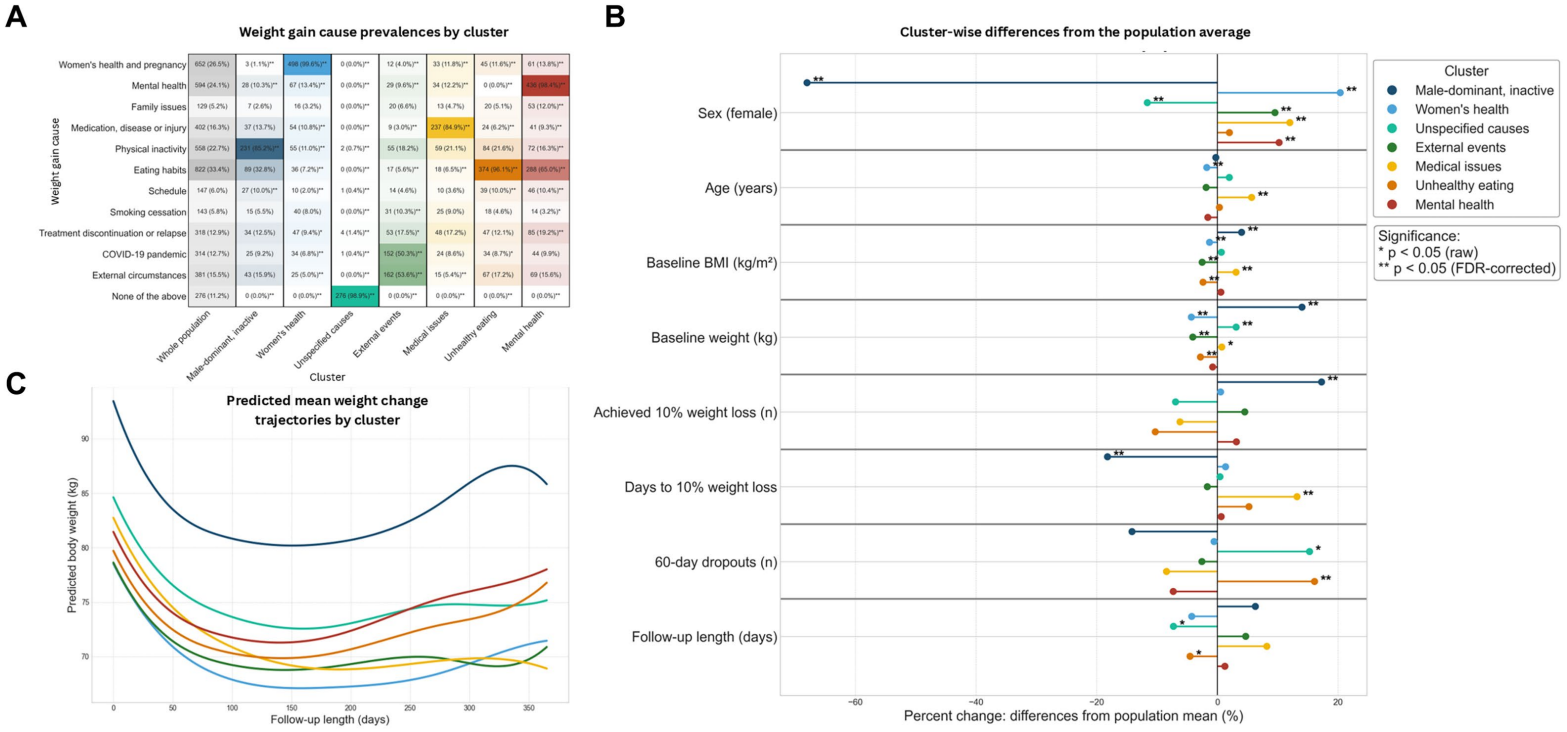
Analysis of weight gain cause-driven behavioral phenotype clusters. **(A)** Indicates the prevalence of each weight gain cause by cluster, with more saturated colors linked to more prevalent variables. **(B)** Shows cluster-wise deviations from the population mean on key demographic and clinical variables expressed as percent changes. *p > 0.05, **p > 0.05 after adjusting for multiple testing. **(C)** Shows mean weight change trajectories per cluster as predicted by linear mixed models.

The seven identified clusters exhibited distinct demographic, behavioral and clinical profiles, which translated into differential treatment adherence and weight loss trajectories (Table 3; Figures 2A,C; Supplementary Figure S3). Clusters characterized by attributing weight gain to physical inactivity—predominantly male patients—showed the highest baseline weight but also the best adherence and weight loss outcomes, including faster achievement of clinically meaningful weight loss (Table 3). In contrast, clusters defined by vague or unspecified weight gain cause reports and mentioning unhealthy eating patterns were associated with higher early dropout rates and poorer weight loss responses. Patients whose weight gain was attributed to medical issues were older, had a higher baseline BMI, and demonstrated slower but sustained weight loss over longer follow-up periods. Clusters defined by reports on women’s health–related factors, external life events or mental health issues displayed intermediate responses, highlighting substantial heterogeneity in treatment dynamics across behavioral phenotypes (Table 3). Linear mixed model analyses indicated a common pattern of rapid initial weight loss during the first 2–3 months of intervention, followed by a plateau and a tendency toward weight regain in most clusters (Figure 2C).

**Table 3:** Comparison of clusters to the population average.

Variable	Population, *N* = 2,463	Male-dominant, inactive, *N* = 271	Women’s health, *N* = 500	Unspecified causes, *N* = 279	External events, *N* = 302	Medical issues, *N* = 279	Unhealthy eating, *N* = 389	Mental health, *N* = 443
Self-reported causes of weight gain								
Women’s health and pregnancy	652 (26.5%)	3 (1.1%)**	498 (99.6%)**	0 (0.0%)**	12 (4.0%)**	33 (11.8%)**	45 (11.6%)**	61 (13.8%)**
Mental health	594 (24.1%)	28 (10.3%)**	67 (13.4%)**	0 (0.0%)**	29 (9.6%)**	34 (12.2%)**	0 (0.0%)**	436 (98.4%)**
Family issues	129 (5.2%)	7 (2.6%)	16 (3.2%)	0 (0.0%)**	20 (6.6%)	13 (4.7%)	20 (5.1%)	53 (12.0%)**
Medication, disease or injury	402 (16.3%)	37 (13.7%)	54 (10.8%)**	0 (0.0%)**	9 (3.0%)**	237 (84.9%)**	24 (6.2%)**	41 (9.3%)**
Physical inactivity	558 (22.7%)	231 (85.2%)**	55 (11.0%)**	2 (0.7%)**	55 (18.2%)	59 (21.1%)	84 (21.6%)	72 (16.3%)**
Eating habits	822 (33.4%)	89 (32.8%)	36 (7.2%)**	0 (0.0%)**	17 (5.6%)**	18 (6.5%)**	374 (96.1%)**	288 (65.0%)**
Schedule	147 (6.0%)	27 (10.0%)**	10 (2.0%)**	1 (0.4%)**	14 (4.6%)	10 (3.6%)	39 (10.0%)**	46 (10.4%)**
Smoking cessation	143 (5.8%)	15 (5.5%)	40 (8.0%)	0 (0.0%)**	31 (10.3%)**	25 (9.0%)	18 (4.6%)	14 (3.2%)*
Treatment discontinuation or relapse	318 (12.9%)	34 (12.5%)	47 (9.4%)*	4 (1.4%)**	53 (17.5%)*	48 (17.2%)	47 (12.1%)	85 (19.2%)**
COVID-19 pandemic	314 (12.7%)	25 (9.2%)	34 (6.8%)**	1 (0.4%)**	152 (50.3%)**	24 (8.6%)	34 (8.7%)*	44 (9.9%)
External circumstances	381 (15.5%)	43 (15.9%)	25 (5.0%)**	0 (0.0%)**	162 (53.6%)**	15 (5.4%)**	67 (17.2%)	69 (15.6%)
None of the above	276 (11.2%)	0 (0.0%)**	0 (0.0%)**	276 (98.9%)**	0 (0.0%)**	0 (0.0%)**	0 (0.0%)**	0 (0.0%)**
Baseline characteristics								
Sex (% of females)	2018 (81.9%)	71 (26.2%)**	493 (98.6%)**	202 (72.4%)**	271 (89.7%)**	256 (91.8%)**	325 (83.5%)	400 (90.3%)**
Age (years)	46.18 ± 10.54	46.05 ± 10.23	45.36 ± 9.87**	47.08 ± 10.54	45.31 ± 11.59	48.79 ± 10.16**	46.33 ± 11.15	45.44 ± 10.12
Baseline BMI (kg/m^2^)	30.06 ± 2.96	31.26 ± 3.25**	29.66 ± 2.72**	30.25 ± 3.18	29.29 ± 2.92**	30.98 ± 2.63**	29.33 ± 2.73**	30.23 ± 2.90
Baseline weight (kg)	82.56 ± 12.25	94.12 ± 14.30**	78.99 ± 9.18**	85.11 ± 14.37**	79.18 ± 11.52**	83.15 ± 10.16*	80.23 ± 11.08**	81.90 ± 10.47
Treatment outcomes								
Total weight loss (%)	−9.21 ± 6.85	−10.25 ± 6.83**	−9.38 ± 6.73	−8.98 ± 6.83	−8.92 ± 6.56	−9.34 ± 7.01	−8.39 ± 7.01**	−9.38 ± 6.91
60-day dropouts (n)	1,080 (43.8%)	102 (37.6%)	218 (43.6%)	141 (50.5%)*	129 (42.7%)	112 (40.1%)	198 (50.9%)**	180 (40.6%)
Achieved 10% weight loss (n)	1,186 (48.2%)	153 (56.5%)**	242 (48.4%)	125 (44.8%)	152 (50.3%)	126 (45.2%)	168 (43.2%)	220 (49.7%)
Days to 10% weight loss	52.59 ± 31.92	43.01 ± 20.12**	53.29 ± 27.56	52.81 ± 31.24	51.70 ± 23.45	59.52 ± 38.64**	55.33 ± 48.45	52.91 ± 26.90
Follow-up length (days)	108.57 ± 111.61	115.39 ± 112.13	103.94 ± 106.06	100.65 ± 113.09*	113.65 ± 110.55	117.46 ± 118.40	103.64 ± 121.72*	109.91 ± 103.10

Data is presented as N (%) for continuous and mean ± SD for categorical variables. Clusters were compared to the population average (Column 2) using Mann–Whitney U test for continuous variables; Chi^2^ test for categorical variables; and Fisher’s exact test for categorical variables with a low expected variance count. *raw *p* > 0.05. **p > 0.05 after adjusting for multiple comparisons using the Benjamini-Hochberg method.

## 4 Discussion

Contemporary advancements in obesity understanding point to a multi-level complexity of weight gain causes and mechanisms (6–8). A number of mainly qualitative studies (9–12) as well as a recent position statement from The Obesity Society (13) highlight the importance of an individual life course perspective in understanding weight gain and obesity. Yet, subjective, patient-reported aspects providing an insider view on weight gain causes have rarely been studied using quantitative methods due to the labor intensity of thematic analysis (19). Recent developments in large language models, however, present an opportunity in bridging that gap by allowing for a standardized and scalable thematic labeling of patient narratives (20, 21). In this paper, we used weight gain cause narratives standardized with GPT4.1 from a large multinational weight loss cohort, with the goal of advancing patient profiling, clinical weight loss prediction and personalized body weight management.

Individual weight gain cause narratives were found to show characteristic associations with sex, age, and to a certain extent, weight loss outcomes and treatment adherence in descriptive analyses and explainable AI predictions. Moreover, direct associations were identified between different weight gain cause themes, representing an interplay of behavioral and life course factors leading to excess weight. To promote the integration of such patterns into clinical obesity management, an unsupervised machine learning-driven patient clustering framework was devised, identifying seven behavioral phenotypes with characteristic demographic, clinical and weight gain cause profiles along with differential treatment responses. Potential individualized modifications to an existing, VLEKT-based clinical weight management framework are discussed for each cluster, primarily focusing on dietary, physical activity and nutritional coaching aspects.

Obesity management encompasses several evidence-based approaches ranging from behavioral modifications influencing dietary, physical activity and lifestyle patterns, pharmacological approaches and bariatric/metabolic surgery (33). Structured multidisciplinary lifestyle interventions consisting of dietary, physical activity and behavioral consulting elements, particularly, interventions based on a VLEKT phase (37, 38) have been shown to produce rapid and substantial weight loss with good safety and tolerability (29, 35, 36). In comparison with pharmacological and surgical approaches, multidisciplinary lifestyle interventions may promote more profound behavior and lifestyle changes needed for long-term weight loss maintenance, nevertheless, the combination of medical nutritional and pharmacological weight loss strategies also holds promise (63). Tailoring components of multidisciplinary interventions to the individual in a personalized and precision weight management framework may further increase treatment effectiveness and adherence (5).

### 4.1 Overview of findings

Over the course of the multidisciplinary weight loss intervention, the study population achieved a notable weight loss of nearly 10% in under 4 months on average. Overall outcomes, as well as subgroup-level treatment response patterns by sex, age and obesity class are in accordance with previous findings regarding the efficacy of the prescribed intervention (29, 35).

Thematic weight gain cause narrative labeling automated using an OpenAI large language model achieved precision, recall and F1 scores of 0.906, 0.897, and 0.897, respectively, evaluated against a human-labeled consensus sample. After standardizing narrative-derived data, clear associations between weight gain causes, age and sex, but not baseline BMI, were identified. Overall, these results suggest that weight gain causes and/or their explanations relate to demographics stronger than to body weight. These findings are in accordance with research identifying a clear demographic stratification of weight gain cause attributions (10, 64) while conflicting results regarding the associations of weight gain narratives and BMI have been reported (64–66).

Older patients more often commented on family issues, medical factors and smoking cessation, while younger individuals mentioned women’s health issues and external circumstances as weight gain causes more frequently. In young women, pregnancy is a common weight gain cause (67), while poor diet quality, a factor more associated with weight gain in the young (68), appears to be related to external circumstances such as increased career, parenting and social life demands in the cohort. At an older age, biological factors such as hormonal changes, injuries, comorbidities and polypharmacy are known causes of weight gain (69).

Among women, besides gestational and menopausal weight gain (67, 70), mental health and family issues are overrepresented. The commonality of mental health-related weight gain cause narratives among women can be partially attributed to both a generally higher prevalence of mental health issues and emotional eating (71, 72) and a greater tendency for psychologically rooted and stress-related weight gain cause explanations (10, 64) among women. Regarding family issues, such as care for or loss of a loved one, a study looking at the associations between bereavement, BMI and demographics found no sex differences (73), but a recent systematic review highlighted increased emotional eating in response to caregiver responsibilities among women during the COVID-19 pandemic (72).

Men, on the other hand, more often referred to unhealthy eating and physical activity habits, mentioned disordered work and meal schedules, or shared vague narratives that the LLM could not fit into any of the established category labels. Other studies looking at weight gain cause attributions from a gender perspective found similar narratives around eating and physical activity behaviors, injuries, alcohol, career changes and work environments more common among men (10, 64).

In terms of the influence of weight gain cause narratives on treatment adherence and outcomes, comparative and predictive analyses suggested that while differential trends prevail among weight gain cause groups, statistically significant associations are sparse. The most consistent patterns were a greater likelihood of achieving 10% weight loss if weight gain was attributed to physical inactivity, with the inverse being true in case of medical issues, such as injuries, comorbidities or weight-inducing drugs, factors that can impose metabolic limitations hindering weight loss (74). Additionally, an increased dropout rate was observed among patients with unspecified weight gain causes, potentially linked to a lower understanding and commitment to change around their body weight (75). As the relevance of individual causalities, or attributions thereof, in treatment outcome predictions, at least in this cohort, appears mixed, observing and interpreting the co-occurrence and interplay of weight-inducing life course factors is necessary.

Taking a step ahead and identifying the interrelationships between self-attributed weight gain causes provides a more nuanced understanding of life course weight development beyond the impact of individual factors. Although many patients reported simple, single-cause explanations, a substantial proportion of the population described multifactorial pathways, which were captured through direct association network and risk ratio analyses. In the network, eating habits and physical inactivity emerged as central hubs influenced by distinct life course and behavioral factors, such as mental health, schedule constraints, medical issues and external circumstances. These patterns highlight how different combinations of distal factors converge on similar weight gain mechanisms, supporting their use in patient profiling and the personalization of behavioral interventions. Similar modular structures linking psychological and lifestyle factors to eating behavior and physical inactivity have been reported elsewhere, reinforcing the relevance of this network-based approach (76).

### 4.2 Behavioral phenotypes and personalized body weight management

To further investigate the translational relevance of weight gain cause narratives in patient profiling and tailored weight management, narrative-derived weight gain cause attributions were integrated with age, sex and baseline BMI data into a behavioral phenotyping framework based on a clustering algorithm. Seven clusters were identified with characteristic demographic, clinical and reported weight gain trajectory profiles as well as distinct treatment response patterns. As such, one cluster with fast, one with slow, three with average and two with limited treatment success were identified. Common patterns of rapid initial body weight loss over 2–3 months followed by a plateau and a tendency toward weight regain were identified in most clusters through linear mixed models. While follow-up data after 4 months was sparse, limiting the generalizability of observations, year-long weight change trajectory modeling further suggests a need for phenotype-specific weight management strategies in the long term.

In this regard, weight gain cause narratives contain several layers of clinically relevant information that can potentially be capitalized upon in weight management settings. First, causal narratives often reveal actionable clinical targets, such as female hormonal transitions, obesogenic drug prescriptions or emotional eating patterns that need to be accounted for in order to achieve holistic health management. Second, not only the content but also the structure of weight gain cause narratives appear relevant. Attributing weight gain to personal responsibility and failure reduced the odds of clinically relevant weight loss success in one study by 38% (17), while understanding and agency felt over excess weight development was associated with a readiness for behavior change in another (75). Ultimately, apart from tailoring weight management to individual circumstances, empathetic listening in itself has a therapeutic effect, as it can help patients make sense of their illness, alleviate its affective burden and reshape their identities and attitudes around weight change (14, 15).

In this sense, the theoretical possibility of integrating patient-informed modifications to an existing, structured multidisciplinary weight loss intervention consisting of a VLEKT, physical activity training and nutritional coaching, was explored. Such modifications, if based on a weight loss intervention with known clinical efficacy (29, 35), may result in a more appropriate, inclusive and effective treatment for patients with different trajectories of excess weight development.

As such, the “Male-dominant, inactive” cluster, consisting mainly of men attributing weight gain to physical inactivity, clinically characterized by baseline obesity, good adherence and fast weight loss, was identified as the best responding cluster. Patients may yield additional long-term benefits from tailoring exercise and coaching interventions in ways that appeal to men, with exercises focusing on functional strength in goal-directed, group-based and competitive settings (77, 78), and dietary protein intake adjusted for higher demands, while ensuring adequate screening for attributions suggesting personal responsibility narratives and internalized weight bias (17, 79, 80).

In contrast, female patients of the “Women’s health” cluster, with baseline overweight and average treatment outcomes, may benefit from a nutritional support of life cycle transitions associated with hormonal changes, increased inflammation and metabolic risk through the inclusion of anti-inflammatory and phytoestrogen-rich items in the diet, such as colorful vegetables and soy products (70, 81–83). The validation and acceptance of biological constraints and family demands can reduce weight shame and self-blame (15).

Patients of the “Unspecified causes” cluster, with baseline obesity and an increased proportion of men, show a high attrition risk. Short follow-ups, high dropouts and vague or unspecific weight gain cause narratives suggest a limited health-related understanding and agency (75), which may benefit from starting the intervention with focused motivational interviewing, reiterated weight history assessment and attainable “commitment contracts” (84–86), with increased check-in frequencies to ensure retention.

In the “External events” profile of mainly women with baseline overweight and average outcomes, weight gain is attributed to externalities that patients have varying levels of control over, ranging from social life, travel and work commitments to a global pandemic. Whatever the case, the weight management team could take into account and prepare for disruptive circumstance changes encouraging patients to leverage portable, ready-made meals and equipment-free exercises such as high-intensity interval training, compatible with travel, unpredictable schedules or extended periods spent indoors (87, 88).

“Medical issues” cluster patients, with baseline obesity and a higher age, mentioned weight gain related to injuries, comorbidities and weight-inducing drugs. Outstanding adherence despite slow weight loss suggests increased health-related motivation, which can be a focus of the intervention: the celebration of non-scale victories can validate age-and comorbidity-related weight loss constraints (29, 35, 74) and emphasize holistic health improvement (15). Consulting with these patients’ general practitioners can achieve obesogenic drug deprescription (89), adequate comorbidity management and referral to a physiotherapist who, in case of injuries, can guide the recommendation of adequate low-impact exercise routines (90).

“Unhealthy eating” cluster patients, with baseline overweight attributed to their eating habits are, again, considered a disengagement risk profile with the lowest mean weight loss, shortest follow-up and highest dropout rates. In their case, emphasizing nutritional education focused on understanding and mitigating unhealthy eating patterns and triggers, screening for comfort eating and food addiction patterns together with internalized weight bias, removing unhealthy items from the household and increasing familiarity with unprocessed food options may yield long-term benefits (17, 91). During initial weight loss, protein replacement VLEKTs appear effective in reducing binge eating and food addiction symptoms (92, 93).

Lastly, patients of the “Mental health” cluster, with baseline overweight, a higher proportion of women and average treatment outcomes, mentioned anxiety, stress, depression, family issues and related emotional eating habits behind their weight gain. First of all, screening for weight-inducing psychiatric drugs is recommended and referral to a mental health expert must be considered if complex trauma or suicidality is suspected (8, 94, 95). Within the scope of a multidisciplinary weight loss intervention, nutritional coaching can focus on emotional eating triggers and the development of alternative mechanisms of coping with emotional distress (96). Nutritional and physical activity approaches proven to alleviate anxious and depressive symptoms, such as ketosis, antiinflammatory foods and supplements, and an “exercise as medicine” framework with enjoyable activities can be leveraged (97–102).

Apart from strictly nutritional strategies such as VLEKT, emerging combined nutritional—pharmacological strategies are paving the way in personalized therapies aimed at achieving long-term, successful weight loss (63, 103). Overall, our results support a view of obesity treatment as a progressive, flexible, and multimodal process in which nutritional strategies could be integrated in a complementary manner with pharmacological treatment for obesity when clinically indicated (32, 63, 104). Within this framework, anti-obesity medications, including newer GLP-1 / GIP agonists, should never be considered a stand-alone therapeutic approach, but rather as a potential facilitator of treatment adherence, appetite control, and the sustainability of behavioral changes, particularly in patient profiles characterized by metabolic limitations, high psychological burden, or suboptimal responses to exclusively behavioral interventions, as reflected in the previously described cluster analyses (32, 104, 105). In particular, patients at high attrition risk, such as the “Unspecified causes” and “Unhealthy eating” clusters in this study, may putatively benefit from adjunct GLP1/GIP agonists to facilitate adherence. Moreover, a clinical study has shown benefits of tailoring drug prescriptions to obesity phenotypes, for example, through administering naltrexone/bupropion for patients with emotional eating as compared to liraglutide for patients with abnormal satiety (41).

Furthermore, the identification of behavioral clusters enables a more precise adaptation of combined nutritional strategies, physical activity recommendations, targeted supplementation, and lifestyle interventions, based on clinically relevant precision personalization criteria such as age, sex, baseline BMI, comorbidities, psychological profile and the life course perspective. Accordingly, the intensity and duration of intervention phases such as the ketogenic phase of a VLEKT (106), the selection of maintenance dietary patterns, the type and volume of exercise, and the level of behavioral or psychotherapeutic support can be tailored to the identified phenotype within multidisciplinary programs (32, 105), with the overarching aim of improving adherence, safety, and the long-term sustainability of weight management in a patient-centered manner.

### 4.3 Strengths, limitations, and future directions

Our study has several notable strengths. First, it addresses the clinical relevance of a pivotal yet largely underexplored dimension of personalized weight management: patient-reported causes of weight gain, capturing the insider perspective of individuals living with obesity in a large, real-world, multinational cohort. By focusing on narrative, self-attributed drivers of weight gain, the study moves beyond traditional biomedical and behavioral variables and incorporates aspects of the life course and psychosocial context that are rarely quantified at scale.

Second, we leveraged a robust and innovative analytical framework combining state-of-the-art large language models with unsupervised machine learning techniques, enabling the scalable transformation of unstructured narrative data into structured, analyzable variables. This approach allowed us to process data from a large, real-world, multinational cohort undergoing a standardized multidisciplinary weight loss intervention, thereby enhancing both the methodological rigor and the external relevance of the findings.

Finally, by addressing a clear gap in the current literature, the study not only explored the role of patient-reported weight gain cause narratives in patient phenotyping and weight loss prediction, but also translated these findings into clinically actionable insights. The identification of weight gain cause narrative–defined patient profiles was directly linked to tailored recommendations for nutrition, physical activity and behavioral coaching, underscoring the potential of this framework to inform more precise, person-centered and practically implementable weight management strategies.

Several limitations should be acknowledged, too. This study represents a secondary analysis of two prospective cohorts originally designed to evaluate a real-world commercial weight loss program; therefore, data collection was not specifically tailored to isolate the causal impact of weight gain attributions on obesity severity or treatment outcomes. Weight gain causes were self-reported lay narratives and may be subject to recall or social desirability bias, although patients’ understanding of their own weight trajectories is itself clinically relevant. While narratives were collected through open-ended questions with no predefined categories, the context of the commercial intervention based on lifestyle modifications may have implicitly favored behavioral explanations reflecting weight stigma over structural causes. The downstream thematic categorization of narratives, while designed to capture a diversity of causes and transparently reported, may have been influenced by authors’ personal biases and be refined further in future studies. In addition, intervention components were partially personalized by design, and detailed data on individual dietary, exercise or coaching recommendations were not available. The specific intervention context, the predominance of women in the cohort, the sparsity of long-term follow-up data and the lack of broader biological and sociodemographic variables may also limit generalizability. This is especially relevant in terms of male-specific patterns, the long-term variability of behaviors, attitudes and resulting clinical outcomes, and the potential emergence of different behavioral phenotypes in different intervention contexts or in light of additional biological and exposome factors.

Future research should apply this AI-driven, patient-centered approach in more structured settings, gender-balanced populations and longer time horizons to further clarify the role of weight gain causal attributions in excess weight development and management. Studies specifically designed to assess weight gain cause narratives in different contexts, the use of advanced and potentially more objective automated thematic analysis frameworks based on multi-agent LLM systems (21), and the parallel identification of not only life course patterns but also attribution styles within narratives may meaningfully contribute to personalized obesity research and care.

## 5 Conclusion

To conclude, patient perspectives on obesity—particularly, narratives describing the causes and trajectories of weight gain—constitute a valuable and largely untapped source of clinical information for obesity phenotyping and the personalization of weight loss interventions. Artificial intelligence offers a powerful means to integrate these unstructured patient-derived insights into data-driven frameworks, enabling their standardization through large language models and their combination with clinical variables in unsupervised clustering approaches. Using data from a large real-world weight loss cohort, we characterized the prevalence, demographic patterns and interrelationships of 12 categories of reported weight gain causes and identified seven distinct weight gain cause-driven patient phenotypes with differential treatment responses. Together, these findings highlight the potential of incorporating patient-reported weight gain narratives into precision, person-centered obesity management and support the development of stratified nutritional, physical activity, behavioral and pharmacological weight loss strategies within established multidisciplinary intervention frameworks.

## Data Availability

The raw data supporting the conclusions of this article, as well as the relevant code, will be made available by the corresponding author upon reasonable request.

## Supplementary material

SUPPLEMENTARY FILE S1 Development and validation of the LLM-based thematic labeling pipeline.

SUPPLEMENTARY DATA S1 Evaluation of LLM-driven thematic labeling of weight gain narratives against a consensus reference on 300 randomly selected samples.

SUPPLEMENTARY DATA S2 Clustering quality evaluation analyses and cluster selection.

SUPPLEMENTARY FIGURE S1 Schematic overview of the data collection and analysis workflow.

SUPPLEMENTARY FIGURE S2 Receiver operating characteristic curve of the weight gain cause-based adjusted random forest prediction of 10% weight loss success.

SUPPLEMENTARY FIGURE S3 Detailed clinical characterization of clusters.
