# Supplementary material for: Integrating patient-reported weight gain cause narratives into personalized obesity management: a data-driven approach with natural language processing and machine learning
Source: Front Nutr. 2026 Apr 14;13:1777240. doi: 10.3389/fnut.2026.1777240 (PMC13123420; doi:10.3389/fnut.2026.1777240)
Supplement: SUPPLEMENTARY DATA S1 — Evaluation of LLM-driven thematic labeling of weight gain narratives against a consensus reference on 300 randomly selected samples. [file Data_Sheet_1.pdf]

S1 File: Development and validation of the LLM-based thematic labeling pipeline

LLM-based weight gain cause categorization and translation prompts

Large language model specifications and prompts for the thematic labeling and English translation of free-text weight gain cause narratives. Instructions and data were sent to OpenAI GPT4.1 and GPT4o-mini models through the application programming interface with a custom Python script.

1. WGC categorization - call parameters:

model: GPT4.1
temperature: 1
max_completion_tokens: 100

1. WGC categorization - system prompt:

You are an expert clinical research assistant specializing in obesity and patient-reported determinants of the disease.
Your task is to accurately classify self-reported reasons for weight gain into a predefined set of English categories.
You read instructions carefully and follow them closely, adhering to the provided category definitions and examples when completing the task.
You understand the complexities and subtleties of patient-reported weight gain reasons, and you are able to classify them into the appropriate categories.
You return a comma-separated list of categories for each input case, without any additional comments or preambles.

1. WGC categorization - user prompt:

You are a meticulous clinical research analyst. Your primary function is to classify patient-reported reasons for weight gain with maximum accuracy, strictly adhering to the rules and definitions provided.
Below, you find the instructions for a research task categorizing patient-reported reasons for weight gain.
Your task is to classify the provided text into one or more of the following categories, based on the predefined category definitions and categorization examples provided.

IMPORTANT INSTRUCTION
The input text you receive may be in various languages (e.g., Spanish, Portuguese, Italian, French, Dutch).
You must contextually understand the input text in its original language and classify it according to the ENGLISH category definitions provided below.
Your output must be in English.

CONTEXT
The patients reporting the reasons for their weight gain are in a structured weight loss treatment program.
Some are undergoing their first intervention cycle. Others have had previous attempts at losing weight, and provide references to their past outcomes and experiences.

Supplementary Material

CATEGORY DEFINITIONS
These categories should be used to classify the reported reasons for weight gain.
In the list below, you will find the definitions and use cases for each category.
Please refer to these instructions carefully when assigning a category or categories to the given case.
Many cases are complex and may require multiple categories to be assigned.
Only assign a category to the weight gain cause if the text explicitly mentions it as a reason for weight gain.
Only use categories that are present in the defined list, do not create novel categories nor use synonyms or alternative syntaxes.

- **womens_health_and_pregnancy**: Weight gain related to pregnancy, postpartum periods and parenthood in general, menopause, fertility treatments, abortions or female-specific hormonal issues.
- **mental_health**: Weight gain related to psychological factors such as anxiety, depression, stress, emotional eating or other mental health and emotional issues.
- **family_issues**: Weight gain related to interpersonal issues, such as caring for sick relatives, relationship or marital problems, loss of a loved one, or other family-related stressors.
- **medication_disease_injury**: Weight gain related to medical conditions, sports or other injuries limiting mobility, or medication side effects.
- **physical_inactivity**: Weight gain related to a reduction of physical activity or exercise. These can be due to both lifestyle changes (e.g., "stopped exercising," "sedentary lifestyle"), medical conditions (e.g., "knee injury") or pandemic-related confinement.
- **eating_habits**: Weight gain related to bad eating habits, such as overeating, emotional and stress eating, snacking, large meal portions, or disordered meal timing.
- **schedule**: Weight gain related to logistical challenges related to work schedules, travel or lack of time disrupting healthy routines.
- **smoking_cessation**: Weight gain related to quitting smoking.
- **treatment_discontinuation_or_relapse**: Weight gain related to the discontinuation of a previous weight loss intervention, a lack of weight maintenance or a relapse after a previous successful weight loss.
- **lifestyle_circumstances**: Weight gain related to life events and circumstances such as a vacation, summer, holidays, moving house, work-related challenges.
- **pandemic**: Weight gain related to the COVID-19 pandemic, lockdowns, isolation or confinement.
- **none_of_above**: Fallback category for cases that do not fit any of the above categories, or when the text is too vague to classify, such as "personal reasons," "bad habits" without detail, or when the response is irrelevant to the cause of the weight gain or is not explicitly stated. Do NOT use this category in combination with any other category, it is a catch-all for cases that do not fit any of the above categories.

EXAMPLES (FEW-SHOT LEARNING)
- **Input**: "hizo parón en sept, aunque en realidad sólo hizo un pedido, no llegó a retomar."
- **Output**: treatment_discontinuation_or_relapse
- **Input**: "lo tuvo que dejar por una enfermedad del marido"
- **Output**: family_issues, treatment_discontinuation_or_relapse
- **Input**: "malos hábitos"
- **Output**: none_of_above
- **Input**: "last year she did the diet and reached 60kg, she has maintained it until summer, now she is very hungry and anxious"
- **Output**: treatment_discontinuation_or_relapse, lifestyle_circumstances, mental_health, eating_habits
- **Input**: "CONFINAMIENTO (cervezas, patatas, ...)
- **Output**: pandemic, eating_habits
- **Input**: "AUMENTOU MUITO NESTES ULTIMOS 2 ANOS PORQUE MUDOU DE TRABALHO PORQUE É MUITO MAIS SEDENTÁRIA. REFERE QUE SE PORTA BEM DURANTE O DIA MAS QUANDO CHEGA A CASA TEM MUITA VONTADE DE COMER.
- **Output**: lifestyle_circumstances, physical_inactivity, eating_habits

- **Input**: "Menopausa, sempre teve tendência para aumentar de peso, refere metabolismo lento, que se adapta facilmente a restrições alimentares, gravidez tardia (mas depois recuperou o peso) e gradualmente foi aumentando.Fez dieta da lev (perdeu, chegou aos 71 kg, mas começou a aumentar e a ter dificuldade a descer, chegou a estar 15 dias a fazer lev e não diminuiu).
- **Output**: womens_health_and_pregnancy, treatment_discontinuation_or_relapse
- **Input**: "TRABALHA (EMPRESA PRÓPRIA) + ESTUDA - POUCO TEMPO, VIDA MAIS SEDENTÁRIA (INTERROMPEU A PRÁTICA REGULAR DE EXERCÍCIO), DESMOTIVOU-SE E DEIXOU DE MONITORIZAR O PESO/O QUE COMIA
- **Output**: lifestyle_circumstances, physical_inactivity, treatment_discontinuation_or_relapse, eating_habits
- **Input**: "TER IDO VIVER PARA ESPANHA DURANTE UM ANO E MEIO. MAIS COMIDA. DESCONTROLO NOS HORÁRIOS DAS REFEIÇÕES.
- **Output**: lifestyle_circumstances, eating_habits, schedule
- **Input**: "Problemas familiares y mucha ansiedad"
- **Output**: family_issues, mental_health
- **Input**: "menopausia, dejar de fumar"
- **Output**: womens_health_and_pregnancy, smoking_cessation
- **Input**: ""Sedentarismo Ansiedad - hambre emocional: comer dulce Malos hábitos alimentarios"
- **Output**: physical_inactivity, mental_health, eating_habits
- **Input**: "menopausia, hipotiroidismo"
- **Output**: womens_health_and_pregnancy, medication_disease_injury
- **Input**: "SE CASO, VIAJES, 2 ABORTOS SEGUIDOS-- ANSIEDAD."
- **Output**: lifestyle_circumstances, womens_health_and_pregnancy, mental_health
- **Input**: "veel op de baan met haar werk"
- **Output**: lifestyle_circumstances
- **Input**: "DA SETTEMBRE GRAVI PROBLEMI FAMILIARI + CURE CON FARMACI ANTIDEPRESSIVI (MOLTO GONFIORE)
- **Output**: family_issues, medication_disease_injury, mental_health"
- **Input**: "tuvo un accidente y cogio peso otra vez"
- **Output**: medication_disease_injury
- **Input**: "desorden horario, come mas por las noches durante el dia se restringe"
- **Output**: schedule, eating_habits
- **Input**: "VERANO"
- **Output**: lifestyle_circumstances
- **Input**: "NO LO SABE PQ DICE QUE SE CUIDA"
- **Output**: none_of_above
- **Input**: "UN POCO TODO"
- **Output**: none_of_above

CRITICAL RULES - YOU MUST FOLLOW THESE EXACTLY:
1. **THE 'none_of_above' RULE**: The category 'none_of_above' is MUTUALLY EXCLUSIVE. If the input text is too vague or does not fit any other category, you MUST return 'none_of_above' AND NOTHING ELSE. You must NEVER assign 'none_of_above' alongside any other category.
2. **NO INFERENCE OR GUESSING**: You MUST only classify causes that are explicitly stated in the text. Do not infer behaviors. For example, if the text says "lost discipline," do not guess this means 'physical_inactivity' or 'eating_habits'; if the text says "social life," do not infer 'eating_habits' unless food or meals are mentioned. The cause must be tangible and mentioned.

TASK
Classify the following text. Return a comma-separated list of applicable English categories from the list provided, without any additional comments or preambles

2. WGC translation - call parameters:

model: GPT4o-mini
temperature: 0.0
max_completion_tokens: 120

2. WGC translation - user prompt:

Translate the following patient-reported reason for their weight gain from its original language (Spanish or Portuguese) to English:
Original text:
English Translation:

2. WGC translation - system prompt:

You are a medical text translator fluent in Romance languages, particularly Spanish and Portuguese. You also speak other European languages.
You understand the subtleties of these languages and can provide clear, natural, and context-aware translations to English, especially in health contexts.
You can handle abbreviations, colloquialisms, or typos effectively.
Your task is to translate patient-reported reasons for weight gain. These texts are from patients with obesity or overweight describing why they believe they gained weight.
Some texts may be vague, complex, abbreviated, simplified, or contain typos.
Preserve the meaning and context accurately.
Output only the English translation, in all lowercases, without any additional comments or preambles.

Example 1:
Original (Spanish): "Estrés en el trabajo y horarios cambiantes "
Translation: "stress at work and changing hours"

Example 2:
Original (Portuguese): "MUDANÇA DE ROTINAS POR ALTERAÇÃO DE TRABALHO
TEVE UM TEMPO SEM TRABALHAR A CUIDAR DE UM FAMILIAR COM INCONSTANCIA ALIMENTAR"

Translation: "change of routines due to job change
spent some time not working taking care of a family member with irregular eating habits"

Pipeline testing and validation

Initial pipeline development used an iterative approach testing different OpenAI models available as of June 2025 with several category labels and prompt structures. Category labels were identified by authors through reading the narratives in the original language and identifying common themes (1). GPT4.1, a model marketed with high instruction adherence (2) was found best performing with a temperature setting of 1 during initial evaluations.

Formal testing was done against a human-labeled reference subset, assessing category-level precision and recall, stability across re-runs, and the impact of temperature and GPT model modifications.

First, 300 randomly selected narratives were labeled with the 12 categories and following the instructions used in the LLM prompt – available above – by three independent, blinded human raters. Interrater agreement was identified as Krippendorff's $\alpha = 0.834$, with category-level α values ranging from 0.556-1.000 (S1 Data). Disagreements were resolved through discussion to establish a 300-item reference matrix (S1 Data). Against this reference, LLM outputs were evaluated quantitatively, assessing category-level and averaged precision, recall and F1 scores, as well as percentage of exact matches. A posterior qualitative audit was realized to assess and quantify the nature of mistakes made by the model, considering debatable, missed, added and switched labels (S1 Data).

GPT4.1 with temperature=1 produced an output with macro-averaged precision, recall and F1 scores of 0.906, 0.897 and 0.897, respectively, with category-level F1 scores in the range of 0.701-1.000 (S1 Data). Qualitative auditing of the LLM-assigned labels revealed that most mistakes are inexact matches, meaning that the LLM recovered part, but not all, of the themes identified by humans in a narrative (S1 Data). Both debatable and missed labels appear in 7.3% of cases, while in 4% of cases, unnecessary or irrelevant labels were added. Complete mismatches were found in 2.3% of cases, with these instances exclusively limited to an incorrect use of the 'none of the above' label.

To ensure the stability across runs using a non-deterministic temperature setting – applied to allow for a more flexible labeling of edge cases –, a cost-effective subset of a 100 narratives were re-labeled with GPT4.1, temperature=1 a total of 5 times. Stability across reruns was evaluated using Krippendorff's alpha treating the five runs with non-deterministic settings as five independent raters, achieving an average alpha of 0.966, with category-level values ranging from 0.905-1.000.

The 300 narratives of the validation set were then re-labeled using a deterministic temperature setting (t=0) in 3 different GPT models to assess pipeline sensitivity: GPT4.1, the flagship instruction following model as of mid-2025 (2); GPT4o-mini, a cost-effective model from 2024 (3); and GPT5.2, OpenAI's flagship model as of early 2026 (4). Reporting macro-averaged precision, recall and F1 scores as well as % of exact matches, our results rank the four tested settings as displayed in S1A Table:

rank	model	temperature	macro-Precision	macro-Recall	macro-F1	% exact match
1	GPT4.1	1	0.906	0.897	0.897	81.0%
2	GPT4.1	0	0.888	0.890	0.883	80.3%
3	GPT5.2	0	0.883	0.888	0.873	75.0%
4	GPT4o-mini	0	0.829	0.862	0.826	64.0%

S1A Table: macro-averaged precision, recall and F1 scores with % of exact matches per GPT model choice evaluated against a reference of 300 samples

Supplementary Material

These results highlight that while different models and temperature settings consistently yield high precision and recall on the same task with F1 scores above 0.8, the percentage of exact match cases varies by model. Data supports the use of larger models for the task, and GPT4.1's high instruction adherence with a slightly increased temperature appears as a valuable approach to achieving high precision and recall while correctly identifying a larger number of edge cases at the same time.

During the time conception and initial analyses of this study in mid-2025 to its publication, large language models underwent constant innovation and progress. To test whether key results and conclusions could be potential artifacts of the labeling pipeline and show sensitivity to model changes, we re-labeled the entire sample of 2463 narratives using GPT5.2, t=0. We found this model's output to be sufficiently distinct from that of GPT4.1 to have relevance in robustness testing, while maintaining a relatively high proportion of exact matches to be considered satisfactory in a clinical context. First, we compared category-level F1 score differences between GPT4.1 and GPT5.2's performance to have an understanding of how GPT5.2-based labels may influence downstream outcomes, then compared research results based on the two engine's labels as per the number of patients reporting each weight gain cause and the size and characteristics of the 7 clusters obtained with the same algorithm as per the main, GPT4.1-based results.

**category**	**GPT4.1 F1**	**GPT5.2 F1**	**Difference of GPT5.2's F1 from GPT4.1's F1**
Women's health and pregnancy	0.965	0.965	0
Mental health	0.975	0.981	+0.006
Family issues	0.914	0.914	0
Medication, disease or injury	0.896	0.912	**+0.016**
Physical inactivity	0.984	0.77	-0.007
Eating habits	0.976	0.919	**-0.057**
Schedule	0.765	0.718	**-0.038**
Smoking cessation	1.000	1.000	0
Treatment discontinuation or relapse	0.789	0.727	**-0.062**
Pandemic	0.985	1.000	**+0.015**
External circumstances	0.701	0.708	+0.007
None of the above	0.81	0.653	**-0.157**

S1B Table: Category-level F1 scores by GPT engine

Accordingly, the None of the above category was found to deviate most between the two models, with deviations also observed at the *'Medication, disease or injury'*, *'Eating habits'*, *'Treatment discontinuation or relapse' and 'Pandemic'* categories. In S1C Table, the number of patients with each label according to engine show the largest differences in the *'Treatment discontinuation or relapse'*, *'Schedule'* and *'External circumstances'* labels, with smaller, although potentially relevant, deviations in the *'Eating habits',*

'Medication, disease or injury' and *'None of the above'* categories. As performance scores in S1A Table indicate that compared with GPT4.1, GPT5.2 suffers greater losses in precision than recall (-0.023 units vs. -0.007 units drop), increases in the number of patients under a given label identified by GPT5.2 are likely driven by false positives.

As per the differences in the number of patients reporting each category, percent change differences per GPT engine were under 5% for eight out of twelve categories; changes of +7.79% and -13.9% were observed in 'Eating habits' and 'External circumstances', respectively, when labeled with GPT5.2; and +25.17% and -27.67% patients were linked to 'Schedule' and 'Treatment discontinuation or relapse' when using the newer engine, generally in line with category-level F1 score differences (S1C Table).

category	GPT4.1	GPT5.2	Difference of GPT5.2 from GPT4.1	Percent change difference
Women's health and pregnancy	652	643	-9	1.38%
Mental health	594	601	+7	1.18%
Family issues	129	129	0	0%
Medication, disease or injury	402	419	+17	+4.23%
Physical inactivity	558	567	+9	+1.61%
Eating habits	822	886	+64	**+7.79%**
Schedule	147	184	+37	**+25.17%**
Smoking cessation	143	145	+2	+1.4%
Treatment discontinuation or relapse	318	230	-88	**-27.67%**
Pandemic	314	313	-1	-0.32%
External circumstances	381	328	-53	**-13.9%**
None of the above	276	289	+13	+4.71%

S1C Table: Number of patients with each label, per GPT engine

To explore pipeline effects more downstream the data analysis pipeline, we recreated the 7 clusters as per the settings applied in the original GPT4.1-based pipeline, however, using GPT5.2-generated labels as inputs alongside age, sex and baseline BMI values. For each regenerated cluster, we considered size, dominant narrative cause or causes, % of women, baseline BMI class and treatment results and adherence relative to the mean as key characteristics, and we compared them to the clusters presented in the main results, built on GPT4.1-added labels (S1D-S1F Tables).

Results of the robustness testing process suggest that most cluster-level patterns are recognized even when changing the large language model serving as the engine of the thematic labeling pipeline, however, some downstream variations of potential clinical relevance did appear in some clusters when using a lower-performing model, highlighting the importance of careful model selection and prompt engineering when building LLM-based clinical research pipelines.

Reference cluster (GPT4.1 labels)	Recreated cluster (GPT5.2 labels)
'Unhealthy eating'<br>n = 389<br>Dominant narrative: 'eating habits' (96%)<br>83% women<br>Baseline overweight<br>Shorter follow-ups<br>Less achievement of 10% weight loss	Cluster 1<br>n = 356<br>Dominant narrative: 'eating habits' (84%)<br>88% women<br>Baseline overweight<br>Shorter follow-ups<br>Less achievement of 10% weight loss
'External events'<br>n = 302<br>Dominant narratives: 'external circumstances' (54%), 'COVID-19 pandemic' (50%), 'physical inactivity', 'treatment discontinuation or relapse' (~18% both)<br>90% women<br>Baseline overweight<br>Longer follow-ups<br>Average weight loss	Cluster 2<br>**n = 250**<br>Dominant narratives: **'COVID-19 pandemic' (73%), 'external circumstances',** 'physical inactivity', 'treatment discontinuation or relapse', 'smoking cessation' (~15% each)<br>90% women<br>**Baseline obesity**<br>Longer follow-ups<br>Average weight loss
'Male-dominant, inactive'<br>n = 271<br>Dominant narratives: 'physical inactivity' (85%), 'eating habits' (33%)<br>26% women<br>Baseline obesity<br>Longer follow-ups<br>Fast weight loss	Cluster 3<br>n = 284<br>Dominant narratives: 'physical inactivity' (71%), **'eating habits' (65%)**<br>21% women<br>Baseline obesity<br>**Average follow-ups**<br>Fast weight loss
'Medical issues'<br>n = 279<br>Dominant narratives: 'medication, disease, injury' (85%), 'physical inactivity' (20%)<br>90 % women<br>Baseline obesity<br>Longer follow-ups<br>Slow weight loss	Cluster 4<br>**n = 322**<br>Dominant narratives: 'medication, disease, injury' (71%), **'physical inactivity' (63%)**<br>90 % women<br>Baseline obesity<br>**Average follow-ups**<br>Slow weight loss
'Unspecified causes'<br>n = 279<br>Dominant narratives: 'none of the above' (99%)<br>72% women<br>Baseline obesity<br>Shorter follow-ups<br>High dropout	Cluster 5<br>n = 295<br>Dominant narratives: 'none of the above' (96%)<br>73% women<br>Baseline obesity<br>Shorter follow-ups<br>High dropout
'Women's health'<br>n = 500<br>Dominant narratives: 'women's health and pregnancy' (100%)<br>99% women<br>Baseline overweight<br>Shorter follow-ups<br>Average weight loss	Cluster 6<br>n = 484<br>Dominant narratives: 'women's health and pregnancy' (99%)<br>99% women<br>Baseline overweight<br>**Average follow-ups**<br>Average weight loss
'Mental health'<br>n = 443<br>Dominant narratives: 'mental health' (98%), 'eating habits' (65%)<br>90% women<br>Baseline obesity<br>Average follow-ups<br>Average weight loss	Cluster 7<br>n = 472<br>Dominant narratives: 'mental health' (97%), 'eating habits' (68%)<br>92% women<br>Baseline overweight<br>Average follow-ups<br>Average weight loss

S1D Table: Comparison of clusters built on GPT4.1 versus GPT5.2-based weight gain cause label

Variable	Population, N=2463	Male-dominant, inactive, N=271	Women's health, N=500	Unspecified causes, N=279	External events, N=302	Medical issues, N=279	Unhealthy eating, N=389	Psyche and eating, N=443
**Self-reported causes of weight gain**								
**Women's health and pregnancy (yes/no)**	652 (26.5%)	3 (1.1%)**	498 (99.6%)**	0 (0.0%)**	12 (4.0%)**	33 (11.8%)**	45 (11.6%)**	61 (13.8%)**
**Mental health (yes/no)**	594 (24.1%)	28 (10.3%)**	67 (13.4%)**	0 (0.0%)**	29 (9.6%)**	34 (12.2%)**	0 (0.0%)**	436 (98.4%)**
**Family issues (yes/no)**	129 (5.2%)	7 (2.6%)	16 (3.2%)	0 (0.0%)**	20 (6.6%)	13 (4.7%)	20 (5.1%)	53 (12.0%)**
**Medication, disease or injury (yes/no)**	402 (16.3%)	37 (13.7%)	54 (10.8%)**	0 (0.0%)**	9 (3.0%)**	237 (84.9%)**	24 (6.2%)**	41 (9.3%)**
**Physical inactivity (yes/no)**	558 (22.7%)	231 (85.2%)**	55 (11.0%)**	2 (0.7%)**	55 (18.2%)	59 (21.1%)	84 (21.6%)	72 (16.3%)**
**Eating habits (yes/no)**	822 (33.4%)	89 (32.8%)	36 (7.2%)**	0 (0.0%)**	17 (5.6%)**	18 (6.5%)**	374 (96.1%)**	288 (65.0%)**
**Schedule (yes/no)**	147 (6.0%)	27 (10.0%)**	10 (2.0%)**	1 (0.4%)**	14 (4.6%)	10 (3.6%)	39 (10.0%)**	46 (10.4%)**
**Smoking cessation (yes/no)**	143 (5.8%)	15 (5.5%)	40 (8.0%)	0 (0.0%)**	31 (10.3%)**	25 (9.0%)	18 (4.6%)	14 (3.2%)*
**Treatment discontinuation or relapse (yes/no)**	318 (12.9%)	34 (12.5%)	47 (9.4%)*	4 (1.4%)**	53 (17.5%)*	48 (17.2%)	47 (12.1%)	85 (19.2%)**
**COVID-19 pandemic (yes/no)**	314 (12.7%)	25 (9.2%)	34 (6.8%)**	1 (0.4%)**	152 (50.3%)**	24 (8.6%)	34 (8.7%)*	44 (9.9%)
**Lifestyle circumstances (yes/no)**	381 (15.5%)	43 (15.9%)	25 (5.0%)**	0 (0.0%)**	162 (53.6%)**	15 (5.4%)**	67 (17.2%)	69 (15.6%)
**None of the above (yes/no)**	276 (11.2%)	0 (0.0%)**	0 (0.0%)**	276 (98.9%)**	0 (0.0%)**	0 (0.0%)**	0 (0.0%)**	0 (0.0%)**
**Baseline characteristics**								
**Sex (% of females)**	2018 (81.9%)	71 (26.2%)**	493 (98.6%)**	202 (72.4%)**	271 (89.7%)**	256 (91.8%)**	325 (83.5%)	400 (90.3%)**
**Age (years)**	46.18 ± 10.54	46.05 ± 10.23	45.36 ± 9.87**	47.08 ± 10.54	45.31 ± 11.59	48.79 ± 10.16**	46.33 ± 11.15	45.44 ± 10.12
**Baseline BMI (kg/m²)**	30.06 ± 2.96	31.26 ± 3.25**	29.66 ± 2.72**	30.25 ± 3.18	29.29 ± 2.92**	30.98 ± 2.63**	29.33 ± 2.73**	30.23 ± 2.90
**Baseline weight (kg)**	82.56 ± 12.25	94.12 ± 14.30**	78.99 ± 9.18**	85.11 ± 14.37**	79.18 ± 11.52**	83.15 ± 10.16*	80.23 ± 11.08**	81.90 ± 10.47
**Treatment outcomes**								
**Total weight loss (%)**	-9.21 ± 6.85	-10.25 ± 6.83**	-9.38 ± 6.73	-8.98 ± 6.83	-8.92 ± 6.56	-9.34 ± 7.01	-8.39 ± 7.01**	-9.38 ± 6.91
**60-day dropouts (n)**	1080 (43.8%)	102 (37.6%)	218 (43.6%)	141 (50.5%)*	129 (42.7%)	112 (40.1%)	198 (50.9%)**	180 (40.6%)
**Achieved 10% weight loss (n)**	1186 (48.2%)	153 (56.5%)**	242 (48.4%)	125 (44.8%)	152 (50.3%)	126 (45.2%)	168 (43.2%)	220 (49.7%)
**Days to 10% weight loss**	52.59 ± 31.92	43.01 ± 20.12**	53.29 ± 27.56	52.81 ± 31.24	51.70 ± 23.45	59.52 ± 38.64**	55.33 ± 48.45	52.91 ± 26.90
**Follow-up length (days)**	108.57 ± 111.61	115.39 ± 112.13	103.94 ± 106.06	100.65 ± 113.09*	113.65 ± 110.55	117.46 ± 118.40	103.64 ± 121.72*	109.91 ± 103.10

S1E Table: Clusters built on GPT4.1-based weight gain cause labels (main results)

Variable	Population, N=2463	Cluster 1, N=356	Cluster 2, N=25	Cluster 3, N=284	Cluster 4, N=322	Cluster 5, N=295	Cluster 6, N=484	Cluster 7, N=472
**Self-reported causes of weight gain**								
**Women's health and pregnancy (yes/no)**	643 (26.1%)	39 (11.0%)**	23 (9.2%)**	6 (2.1%)**	19 (5.9%)**	6 (2.0%)**	483 (99.8%)**	67 (14.2%)**
**Mental health (yes/no)**	601 (24.4%)	0 (0.0%)**	21 (8.4%)**	37 (13.0%)**	30 (9.3%)**	1 (0.3%)**	56 (11.6%)**	456 (96.6%)**
**Family issues (yes/no)**	129 (5.2%)	12 (3.4%)	19 (7.6%)	7 (2.5%)	11 (3.4%)	5 (1.7%)**	16 (3.3%)	59 (12.5%)**
**Medication, disease or injury (yes/no)**	419 (17.0%)	22 (6.2%)**	19 (7.6%)**	28 (9.9%)**	228 (70.8%)**	1 (0.3%)**	66 (13.6%)	55 (11.7%)**
**Physical inactivity (yes/no)**	567 (23.0%)	26 (7.3%)**	36 (14.4%)**	203 (71.5%)**	202 (62.7%)**	1 (0.3%)**	37 (7.6%)**	62 (13.1%)**
**Eating habits (yes/no)**	886 (36.0%)	298 (83.7%)**	23 (9.2%)**	185 (65.1%)**	23 (7.1%)**	0 (0.0%)**	38 (7.9%)**	319 (67.6%)**
**Schedule (yes/no)**	184 (7.5%)	49 (13.8%)**	11 (4.4%)	46 (16.2%)**	10 (3.1%)**	2 (0.7%)**	17 (3.5%)**	49 (10.4%)*
**Smoking cessation (yes/no)**	145 (5.9%)	30 (8.4%)	36 (14.4%)**	16 (5.6%)	6 (1.9%)**	3 (1.0%)**	35 (7.2%)	19 (4.0%)
**Treatment discontinuation or relapse (yes/no)**	230 (9.3%)	34 (9.6%)	38 (15.2%)**	29 (10.2%)	27 (8.4%)	7 (2.4%)**	36 (7.4%)	59 (12.5%)*
**COVID-19 pandemic (yes/no)**	313 (12.7%)	31 (8.7%)*	182 (72.8%)**	26 (9.2%)	16 (5.0%)**	1 (0.3%)**	17 (3.5%)**	40 (8.5%)**
**Lifestyle circumstances (yes/no)**	328 (13.3%)	65 (18.3%)**	42 (16.8%)	54 (19.0%)**	42 (13.0%)	6 (2.0%)**	33 (6.8%)**	86 (18.2%)**
**None of the above (yes/no)**	289 (11.7%)	1 (0.3%)**	0 (0.0%)**	0 (0.0%)**	0 (0.0%)**	282 (95.6%)**	6 (1.2%)**	0 (0.0%)**
**Baseline characteristics**								
**Sex (% of females)**	2018 (81.9%)	315 (88.5%)**	225 (90.0%)**	59 (20.8%)**	291 (90.4%)**	215 (72.9%)**	477 (98.6%)**	436 (92.4%)**
**Age (years)**	46.18 (±10.54)	46.63 (±10.83)	47.85 (±11.02)**	46.64 (±10.40)	46.58 (±11.33)	46.82 (±10.73)	45.14 (±9.86)**	45.06 (±9.94)**
**Baseline BMI (kg/m²)**	30.06 (±2.96)	28.53 (±2.67)**	31.17 (±2.56)**	31.42 (±3.19)**	30.15 (±2.84)	30.40 (±3.16)	29.80 (±2.67)	29.78 (±2.83)
**Baseline weight (kg)**	82.56 (±12.25)	77.32 (±10.44)**	83.93 (±9.91)**	94.98 (±13.49)**	81.83 (±11.89)	85.39 (±14.21)**	79.23 (±9.01)**	80.47 (±10.00)**
**Treatment outcomes**								
**Total weight loss (%)**	-9.21 (±6.85)	-8.28 (±7.20)**	-9.58 (±7.16)	-9.51 (±6.86)	-9.45 (±6.64)	-8.94 (±6.66)	-9.61 (±6.64)	-9.15 (±6.85)
**60-day dropouts (n)**	1186 (48.2%)	149 (41.9%)**	123 (49.2%)	153 (53.9%)	154 (47.8%)	131 (44.4%)	244 (50.4%)	232 (49.2%)
**Achieved 10% weight loss (n)**	52.59 (±31.92)	50.66 (±25.75)	53.47 (±22.83)	45.75 (±46.43)**	56.99 (±36.70)	54.79 (±33.54)	53.25 (±27.48)	53.02 (±27.18)
**Days to 10% weight loss**	1080 (43.8%)	174 (48.9%)	105 (42.0%)	119 (41.9%)	123 (38.2%)	153 (51.9%)**	211 (43.6%)	195 (41.3%)
**Follow-up length (days)**	108.57 (±111.61)	105.44 (±120.27)	122.14 (±121.48)	108.75 (±113.87)	109.40 (±100.12)	100.20 (±111.88)**	108.07 (±114.08)	108.84 (±102.35)

S1F Table: Clusters built on GPT5.2-based weight gain cause labels (validation results)

S1 File References

1. Braun V, Clarke V. Using thematic analysis in psychology. *Qual Res Psychol* (2006) 3:77–101. doi: 10.1191/1478088706qp063oa

2. Introducing GPT-4.1 in the API. https://openai.com/index/gpt-4-1/. [Accessed December 3, 2025]

3. GPT-4o mini: advancing cost-efficient intelligence. https://openai.com/index/gpt-4o-mini-advancing-cost-efficient-intelligence/ [Accessed February 17, 2026]

4. GPT-5 is here. https://openai.com/gpt-5/ [Accessed February 17, 2026]
