# Supplementary material for: Integrating patient-reported weight gain cause narratives into personalized obesity management: a data-driven approach with natural language processing and machine learning
Source: Front Nutr. 2026 Apr 14;13:1777240. doi: 10.3389/fnut.2026.1777240 (PMC13123420; doi:10.3389/fnut.2026.1777240)
Supplement: SUPPLEMENTARY DATA S2 — Clustering quality evaluation analyses and cluster selection. [file Data_Sheet_2.docx]

**S1 Figure**: Schematic overview of the data collection and analysis workflow.

**S2 Figure**: Receiver operating characteristic curve of the weight gain cause-based adjusted random forest prediction of 10% weight loss success.

**S3 Figure**: Detailed clinical characterization of clusters. Demographic, baseline body weight and treatment response characteristics of clusters were compared to the population mean. P-values were calculated with a Mann-Whitney U test for continuous and a Chi2 or Fisher’s exact test for categorical variables, and adjusted for multiple comparisons using the Benjamini-Hochberg method.
