# Supplementary figures and images for: Integrating patient-reported weight gain cause narratives into personalized obesity management: a data-driven approach with natural language processing and machine learning
Source: Front Nutr. 2026 Apr 14;13:1777240. doi: 10.3389/fnut.2026.1777240 (PMC13123420; doi:10.3389/fnut.2026.1777240)

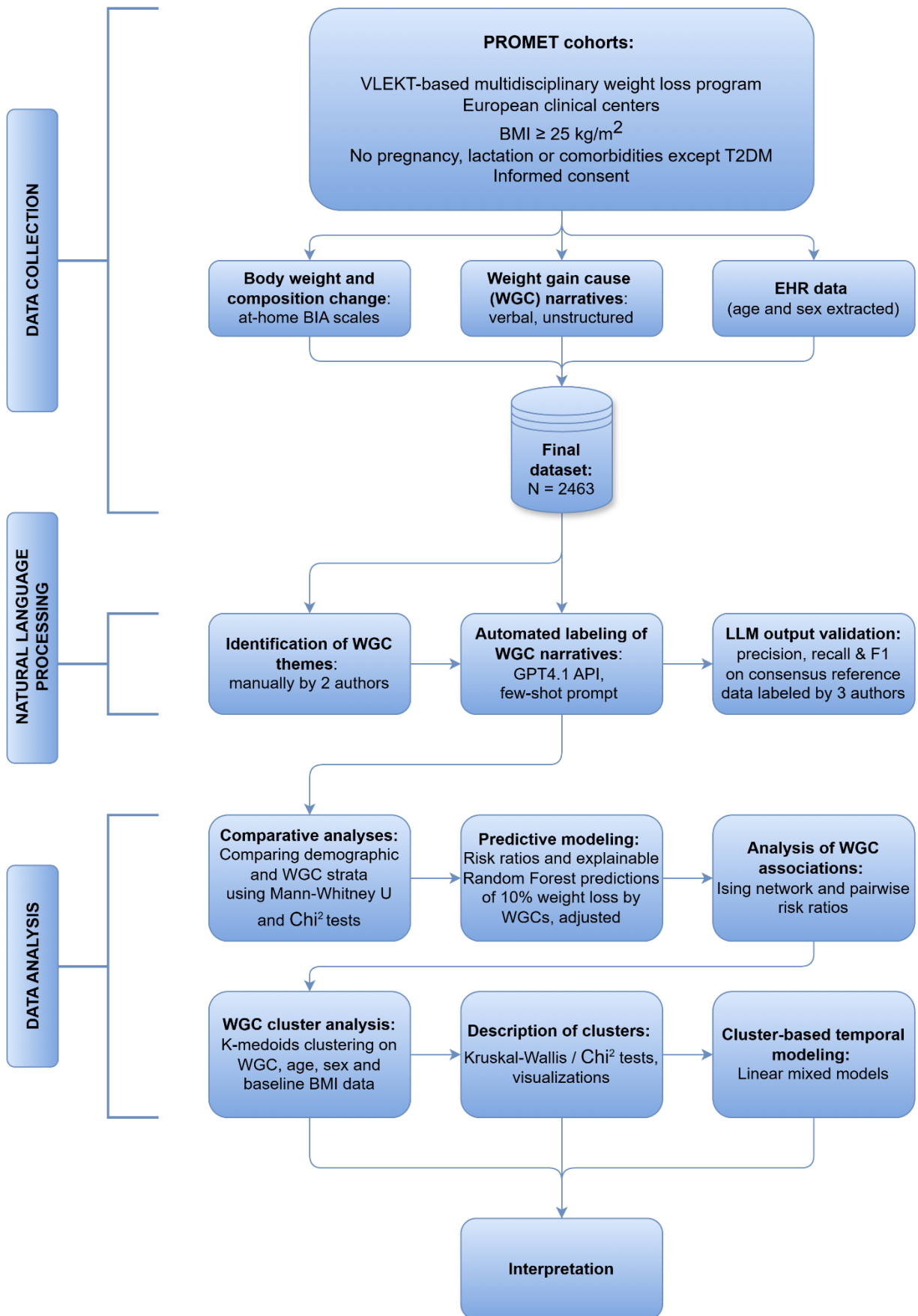

Supplement: SUPPLEMENTARY FIGURE S1 — Schematic overview of the data collection and analysis workflow. [file Image_1.pdf]

10% weight loss achievement by WGCs, adjusted for age, sex and baseline BMI: ROC Curve

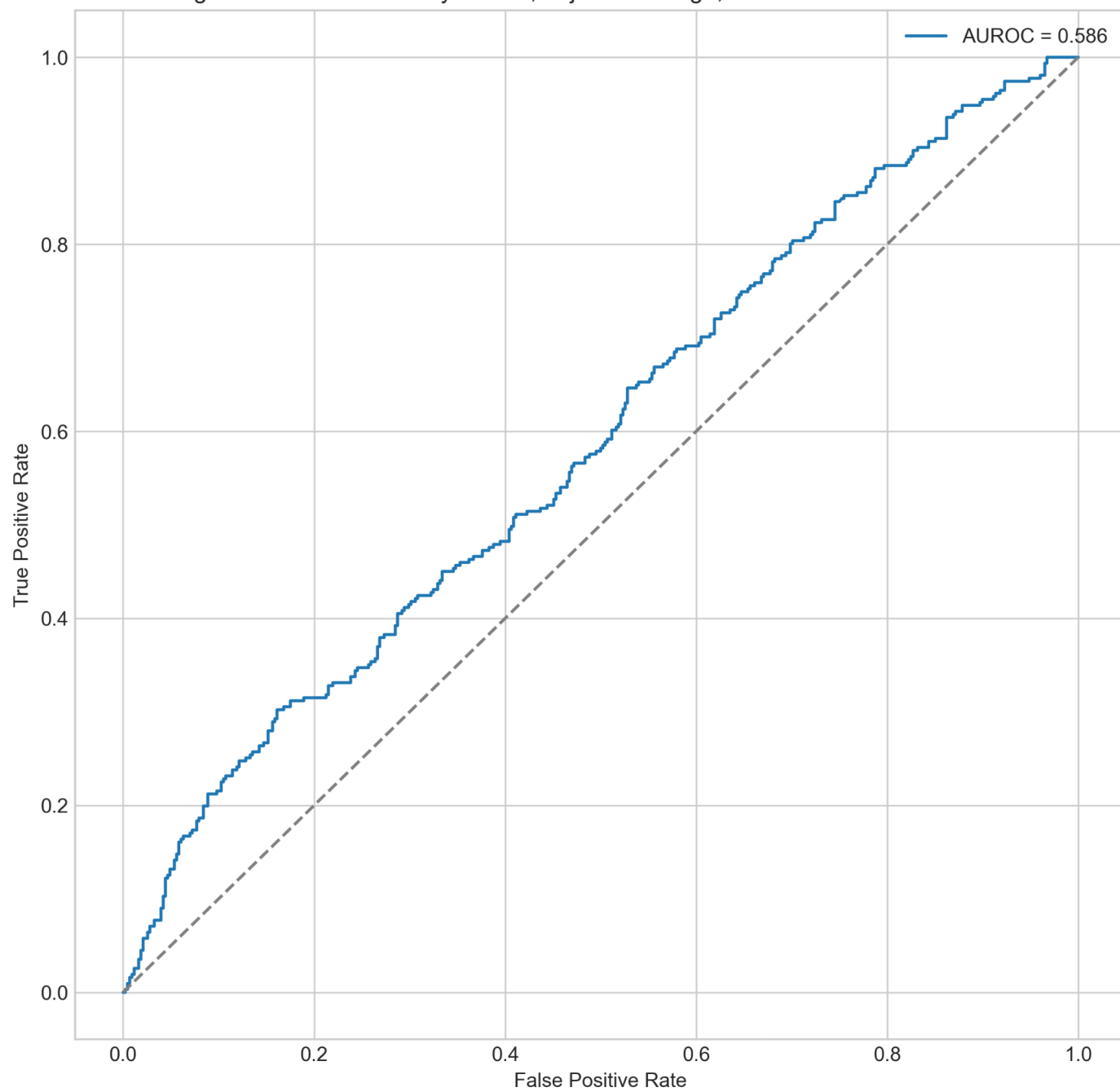

Supplement: SUPPLEMENTARY FIGURE S2 — Receiver operating characteristic curve of the weight gain cause-based adjusted random forest prediction of 10% weight loss success. [file Image_2.pdf]

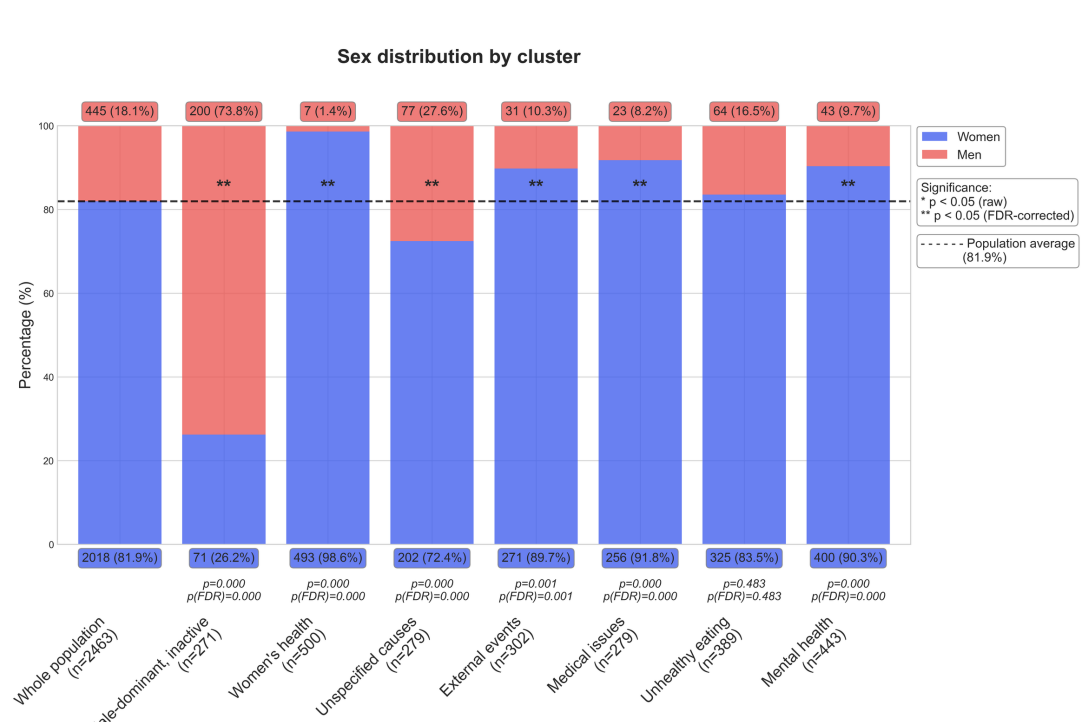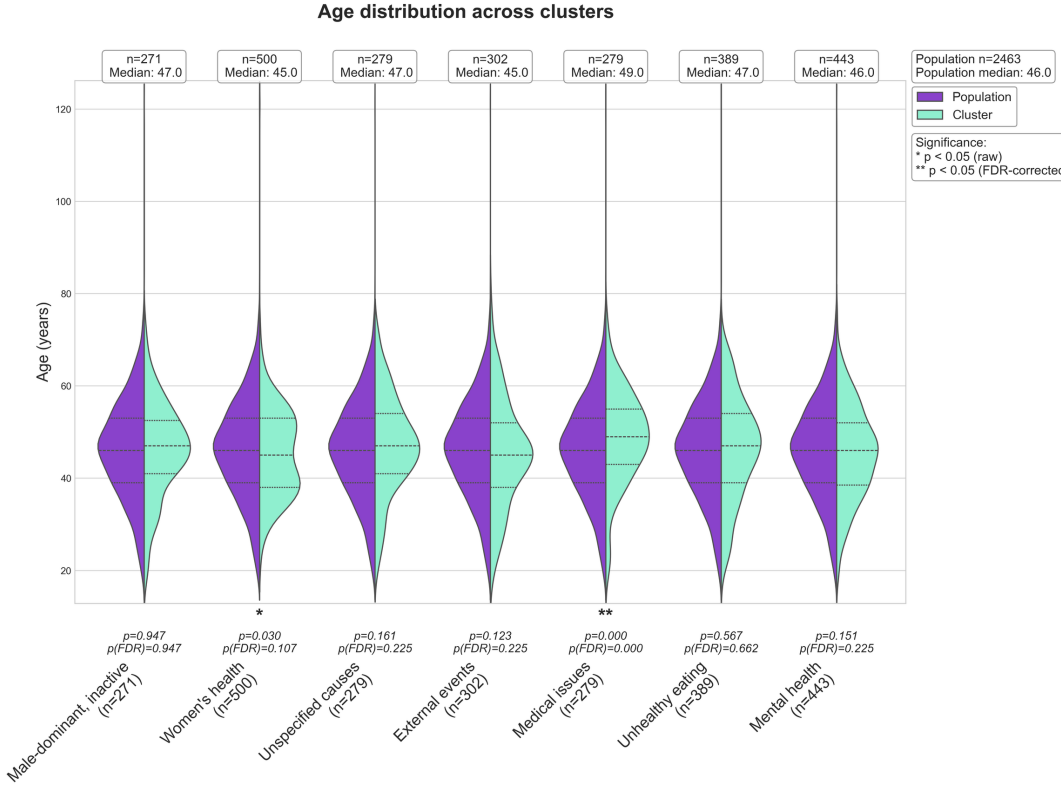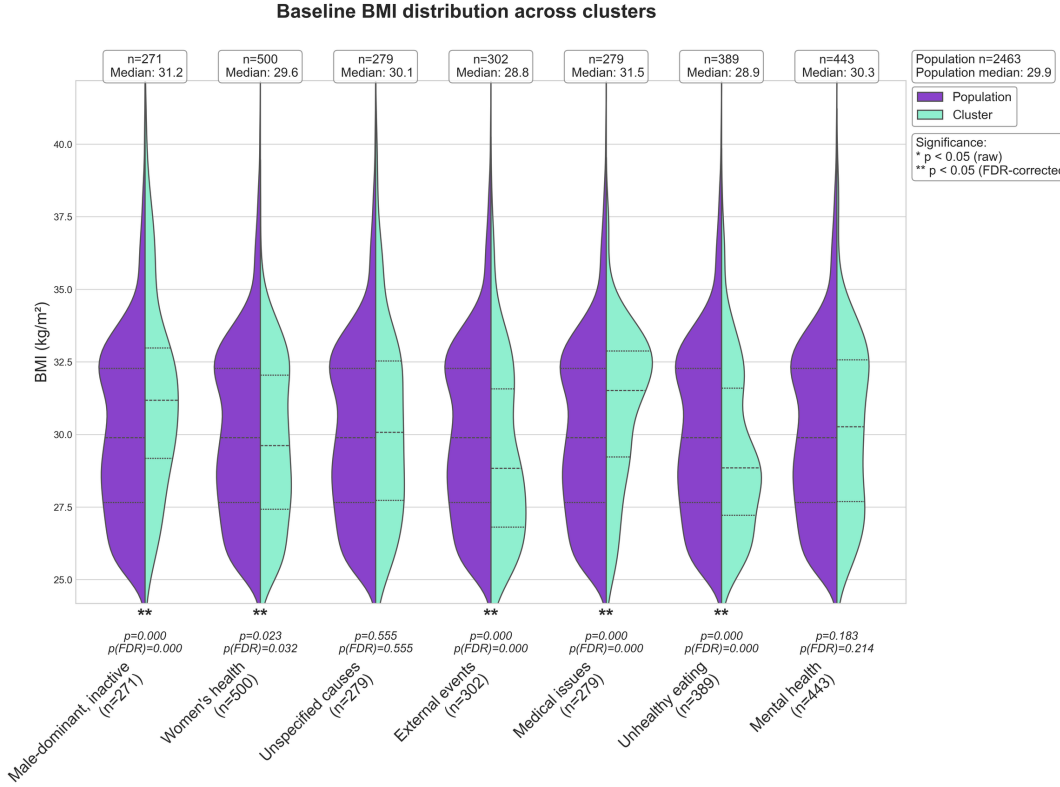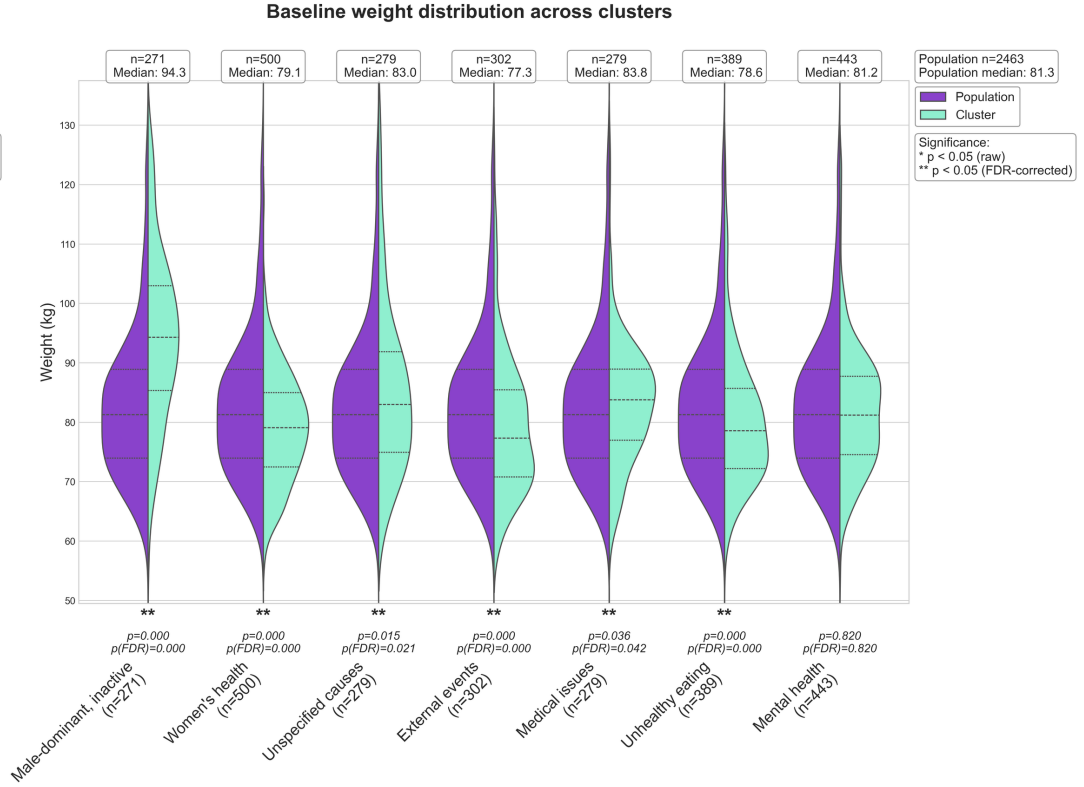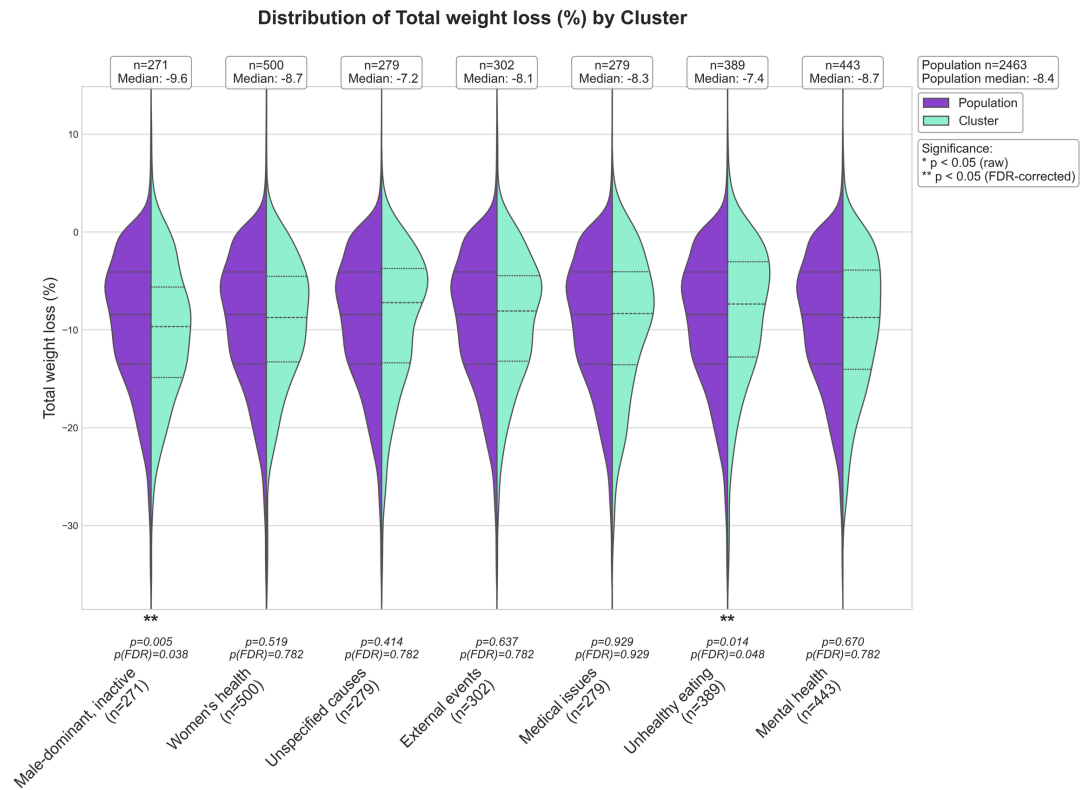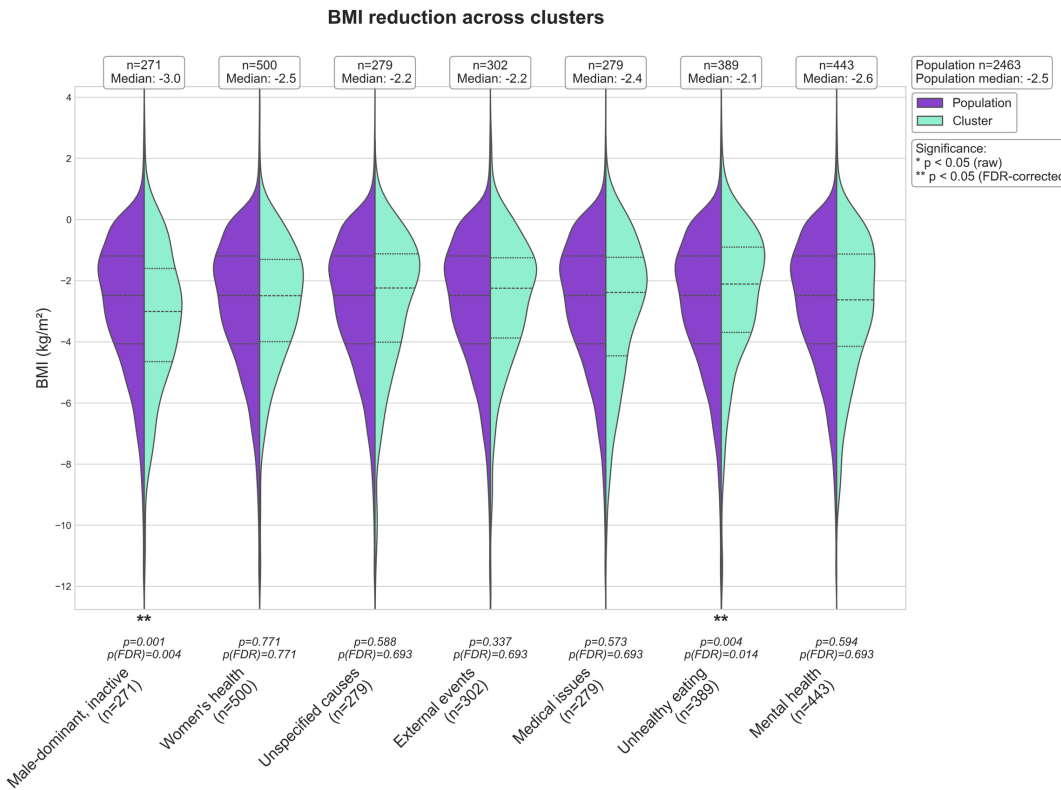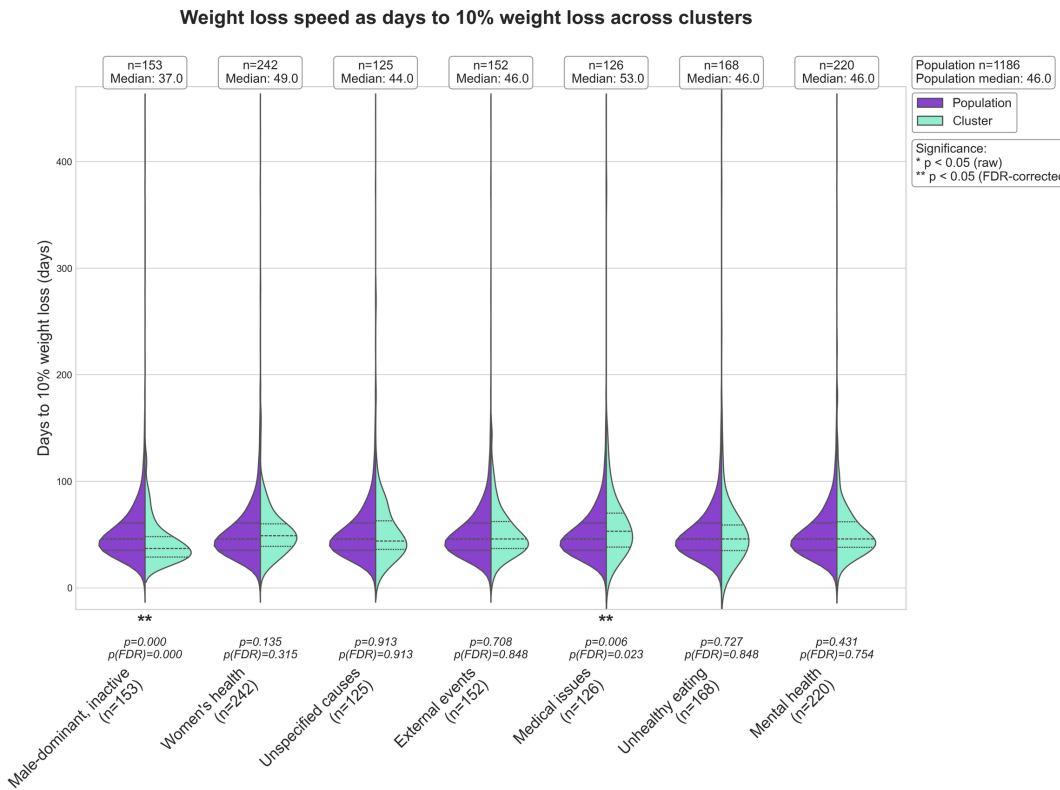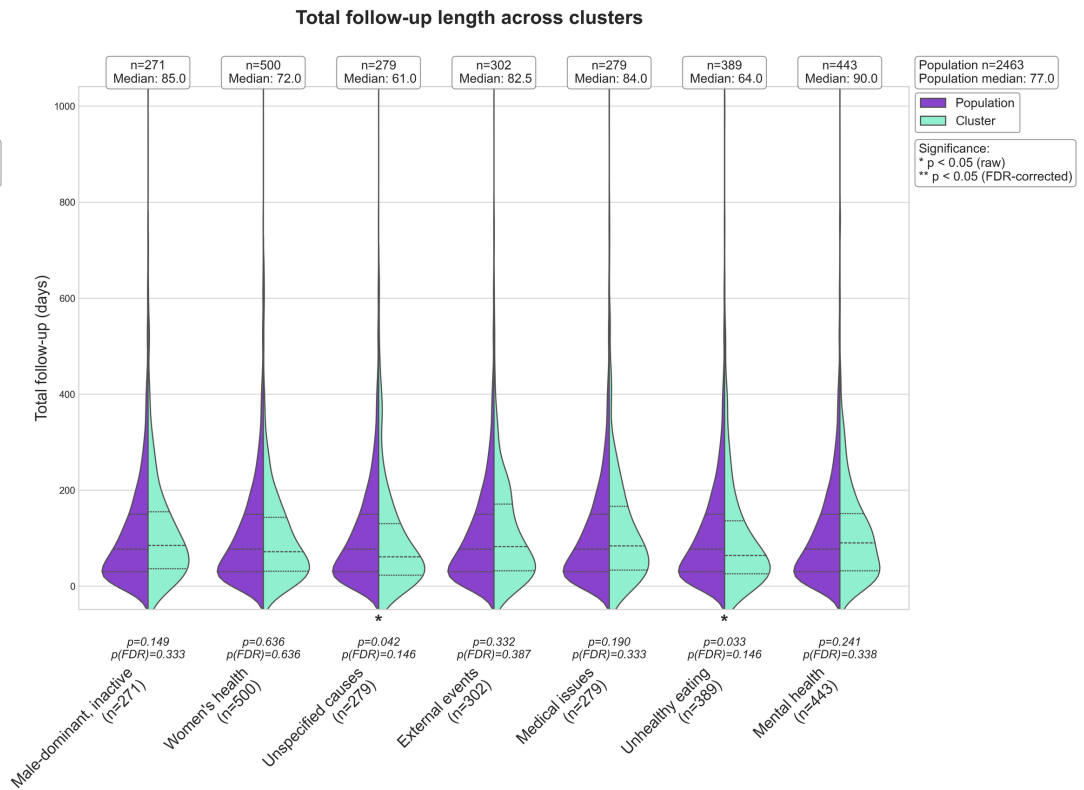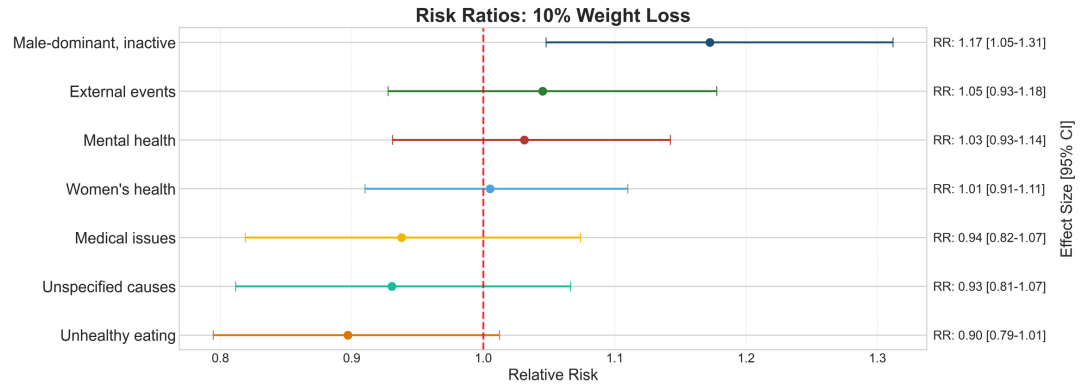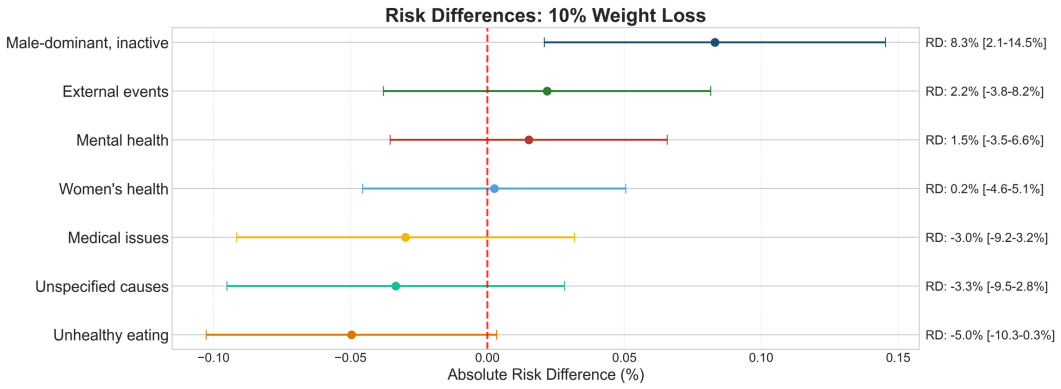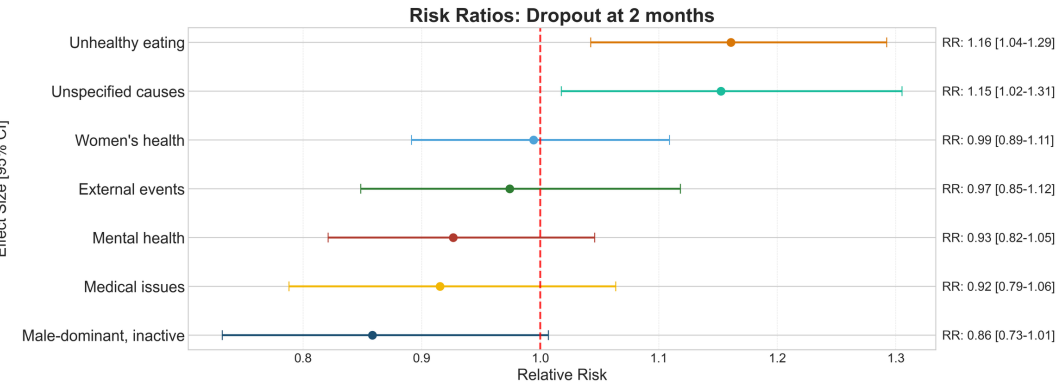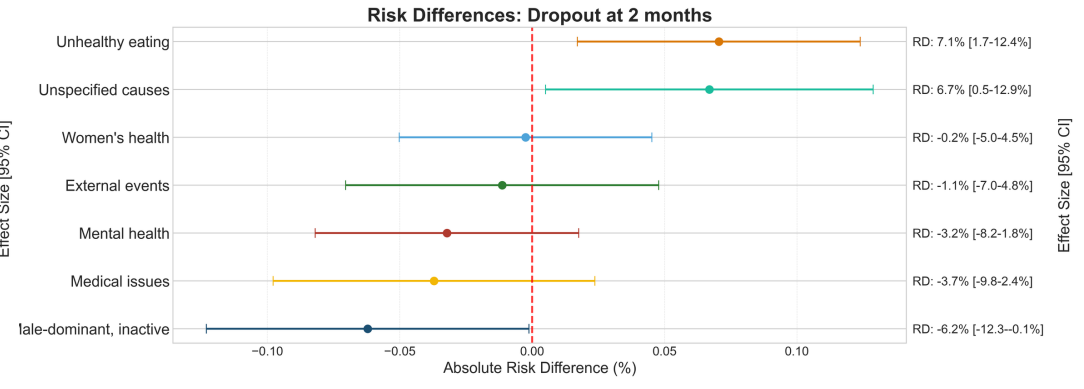

Supplement: SUPPLEMENTARY FIGURE S3 — Detailed clinical characterization of clusters. [file Image_3.pdf]
